# Novel endoscopic techniques for the diagnosis of gastric *Helicobacter pylori* infection: a systematic review and network meta-analysis

**DOI:** 10.3389/fmicb.2024.1377541

**Published:** 2024-08-26

**Authors:** Wenzhe Hao, Lin Huang, Xuejun Li, Hongyu Jia

**Affiliations:** ^1^The Graduated School, Anhui University of Chinese Medicine, Hefei, China; ^2^Department of Gastroenterology, The Second Affiliated Hospital of Anhui University of Chinese Medicine, Hefei, China; ^3^School of Public Health, Anhui Medical University, Hefei, China

**Keywords:** novel endoscopic techniques, gastric, *Helicobacter pylori* infection, meta-analysis, diagnosis

## Abstract

**Objective:**

This study aimed to conduct a network meta-analysis to compare the diagnostic efficacy of diverse novel endoscopic techniques for detecting gastric *Helicobacter pylori* infection.

**Methods:**

From inception to August 2023, literature was systematically searched across Pubmed, Embase, and Web of Science databases. Cochrane’s risk of bias tool assessed the methodological quality of the included studies. Data analysis was conducted using the R software, employing a ranking chart to determine the most effective diagnostic method comprehensively. Convergence analysis was performed to assess the stability of the results.

**Results:**

The study encompassed 36 articles comprising 54 observational studies, investigating 14 novel endoscopic techniques and involving 7,230 patients diagnosed with gastric *H. pylori* infection. Compared with the gold standard, the comprehensive network meta-analysis revealed the superior diagnostic performance of two new endoscopic techniques, Magnifying blue laser imaging endoscopy (M-BLI) and high-definition magnifying endoscopy with i-scan (M-I-SCAN). Specifically, M-BLI demonstrated the highest ranking in both sensitivity (SE) and positive predictive value (PPV), ranking second in negative predictive value (NPV) and fourth in specificity (SP). M-I-SCAN secured the top position in NPV, third in SE and SP, and fifth in PPV.

**Conclusion:**

After thoroughly analyzing the ranking chart, we conclude that M-BLI and M-I-SCAN stand out as the most suitable new endoscopic techniques for diagnosing gastric *H. pylori* infection.

**Systematic review registration:**

https://inplasy.com/inplasy-2023-11-0051/, identifier INPLASY2023110051.

## 1 Introduction

*Helicobacter pylori* is a Gram-negative bacterium infecting the epithelial layer of the human stomach, capable of colonizing and persisting in a unique biological niche within the gastric lumen (Correa, 1988; Dunn et al., 1997). In 1994, the International Agency for Research on Cancer (IARC) classified *H. pylori* as a Group I carcinogen (IARC, 1994). It is linked to chronic gastritis, gastric ulcers, duodenal ulcers, gastric adenocarcinoma, and gastric mucosa-associated lymphoid tissue lymphoma (Suerbaum and Michetti, 2002; Graham, 2015). Over half of the world’s population is infected, with prevalence reaching 25 to 50% in developed countries and 70 to 90% in developing nations (Xia and Talley, 1997; Hooi et al., 2017). Post-infection, it sequentially leads to chronic atrophic gastritis, intestinal metaplasia, dysplasia, and gastric cancer (Correa, 1988). Timely diagnosis holds immense significance for *H. pylori* eradication, preventing diseases such as gastric cancer (Choi et al., 2018).

The diagnosis of gastric *H. pylori* infection traditionally involves invasive techniques like histological examination, *H. pylori* culture, and polymerase chain reaction, as well as non-invasive methods such as serological detection, urea breath test (UBT), and fecal antigen detection (Vaira et al., 2002; Wang et al., 2015; Choi et al., 2018). However, the accuracy of invasive detection is affected by factors like biopsy location, size, number, staining methods, and antibiotic use. On the other hand, non-invasive techniques can be influenced by antibiotics, bismuth, and test reaction time (Logan and Walker, 2001). Are there more accurate and intuitive diagnostic options for gastric *H. pylori* infection? Over the past decade, plain white light imaging endoscopy (WLE) has been utilized as a diagnostic tool for the invasive detection of gastric *H. pylori* infection. While WLE cannot replace UBT as the diagnostic foundation, it can determine the presence or absence of *H. pylori* infection during primary disease examination. WLE offers advantages in intuitiveness, immediacy, strong operability, and the potential to avoid biopsy. It guides follow-up examination and treatment, presenting a novel approach to *H. pylori* diagnosis (Glover et al., 2020b). In this context, endoscopic invasive methods for diagnosing gastric *H. pylori* infection have emerged as a superior screening tool and research focus. Recent advancements in endoscopic technology have introduced new types of endoscopy, surpassing traditional WLE. These include Magnifying Endoscopy (ME), Narrow Band Imaging Endoscopy (NBI), Linked Color Imaging Endoscopy (LCI), Confocal Laser Endomicroscopy (CLE), Near-Infrared Raman Spectroscopy Endoscopy (NIR), Artificial Intelligence-based Computer-Aided Diagnosis (AI-CAD), and Convolutional Neural Network (AI-CNN; Glover et al., 2020b). Compared to traditional endoscopy, the various images produced by these new endoscopic methods enable better observation of microscopic structures, such as gastric pit patterns, microvessels, cell morphology, and even microorganisms.

Additionally, AI combined with endoscopic images can be trained to determine the presence or absence of infection. The enlargement of gastric pits, disappearance of collecting veins, and the vanishing capillary network increasingly indicate specific endoscopic characteristics of gastric *H. pylori* infection. This facilitates rapid and minimally invasive endoscopic diagnosis, bringing it closer to pathological diagnosis (Ji and Li, 2014).

A prospective study conducted by Gonen et al. in Turkey, involving 129 patients, affirmed the superiority of high-resolution Magnifying Endoscopy (ME) over White Light Endoscopy (WLE) in diagnosing gastric gastritis associated with *H. pylori* infection (Gonen et al., 2009). In another prospective study by Ozgur et al. (2013), it was demonstrated that mucosal changes in patients with gastric *H. pylori* infection were more readily identified using narrow-band imaging (NBI) compared to WLE, with NBI exhibiting a high sensitivity of 92.86% (Özgür et al., 2015). Yagi et al. (2014) compared the diagnostic efficacy of WLE and Magnifying NBI (M-NBI) in patients with post-endoscopic resection. The interobserver agreement for conventional endoscopy was moderate (0.56), with sensitivity and specificity at 79 and 52%, respectively. In contrast, M-NBI demonstrated substantial interobserver agreement (0.77), with sensitivity and specificity reaching 91 and 83% (Yagi et al., 2014). Qi et al. compared ME and M-I-Scan’s diagnostic performance and image quality for gastric *H. pylori* infection. M-I-Scan exhibited high sensitivity and specificity, surpassing ME specificity significantly (sensitivity: 95.45% vs. 95.45%, specificity: 93.55% vs. 80.65%; Qi et al., 2013). In 2017, Shichijo et al. developed an Artificial Intelligence-based Convolutional Neural Network (AI-CNN) capable of diagnosing gastric *H. pylori* infection through endoscopic images. After learning from 32,208 images across 1750 patients, AI-CNN demonstrated higher accuracy, specificity, and sensitivity than 23 endoscopists. Additionally, the time required for AI-CNN to generate diagnoses was considerably faster than that of the endoscopists (194 s vs. 230 min; Shichijo et al., 2017). Simultaneously, Itoh et al. demonstrated in their study that their AI-CNN deep learning algorithm, trained on 149 endoscopic images under WLE of patients with *H. pylori* status, achieved diagnostic sensitivity and specificity of 86.7% each when tested on 30 new endoscopic photos (Itoh et al., 2018). In 2023, Zhang et al. published research unveiling AI-WLE, developed using 47,239 images from 1826 patients, which exhibited an accuracy of 91.1% [95% confidence interval (CI): 85.7–94.6]. This accuracy was significantly higher than endoscopists (15.5% [95% CI: 9.7–21.3%]). Furthermore, its high sensitivity (0.9290) and specificity (0.8930) were confirmed (Zhang et al., 2023).

In detecting gastric *H. pylori* infection, different new endoscopes exhibit varying characteristics in terms of sensitivity, specificity, and diagnostic efficiency. Existing systematic reviews or meta-analyses have primarily focused on comparing non-magnifying endoscopy or artificial intelligence for diagnosing human gastric *H. pylori* images, with a notable absence of comparisons among different new endoscopic techniques. Consequently, evidence-based recommendations regarding the most suitable diagnostic method for gastric *H. pylori* infection still need to be made (Qi et al., 2016; Bang et al., 2020; Glover et al., 2020a,b). Hence, it is crucial to identify an appropriate technique for diagnosing gastric *H. pylori* infection among the array of new endoscopic options, particularly when clinicians select different endoscopes for patient diagnosis in clinical practice.

Network meta-analysis, a contemporary evidence-based technique utilizing direct or indirect comparisons, is employed to assess the effects of multiple interventions on disease and estimate the hierarchical order of each intervention (Rouse et al., 2017). In this study, we aggregated existing evidence. We conducted a network meta-analysis to compare novel endoscopic techniques (BLI, LCI, CLE, NBI, ME, AI-CNN, etc.) to evaluate and contrast their diagnostic performance in gastric *H. pylori* infection patients. This approach aims to furnish patients and clinicians with disease-specific, evidence-based data, facilitating the selection of suitable diagnostic methods for screening and diagnosis.

## 2 Materials and methods

The study adhered rigorously to the Preferred Reporting Items for Systematic Reviews and Meta-Analyses guidelines. The protocol has been registered on the INPLASSY website with the registration number INPLASY2023110051.

### 2.1 Search strategy

We systematically searched three electronic databases (PubMed, Embase, and Web of Science) for data spanning from the databases inception to August 2023. The search strategy aligned with the PICOS tool (Schardt et al., 2007): (P) population—encompassing positive and negative patients with gastric *H. pylori* infection; (I) intervention—involving novel endoscopic techniques; (C) control group—comprising gold standard detection methods for gastric *H. pylori* infection such as RUT, UBT test, or other endoscopic techniques; (O) Results—entailing a comprehensive assessment of the predictive value of novel endoscopic techniques in patients with positive gastric *H. pylori* infection, considering sensitivity, specificity, positive predictive value, and negative predictive value; (S) Type of study—focused on observational studies. The detailed search strategy is outlined in Table 1 (using the PubMed database as an example). Embase and Web of Science databases are searched in the Supplementary material.

**Table 1:** Search strategy on PubMed.

#1	*Helicobacter pylori*[MeSH Terms]
#2	(((*Helicobacter nemestrinae*) OR (*Campylobacter pylori*)) OR (*Campylobacter pylori* subsp. pylori)) OR (*Campylobacter pylori*dis)
#3	(#1) OR (#2)
#4	Magnifying endoscope
#5	Narrow Band Imaging[MeSH Terms]
#6	((((((((Band Imaging, Narrow) OR (Band Imagings, Narrow)) OR (Imaging, Narrow Band)) OR (Imagings, Narrow Band)) OR (Narrow Band Imagings)) OR (Narrowband Imaging)) OR (Imaging, Narrowband)) OR (Imagings, Narrowband)) OR (Narrowband Imagings)
#7	(#5) OR (#6)
#8	Blue laser imaging
#9	Linked Color Imaging
#10	Fuji Intelligent Chromo Endoscopy
#11	(Iscan) OR (I-Scan)
#12	Microscopy, Confocal[MeSH Terms]
#13	((((((((((((((((Confocal Microscopy) OR (Confocal Microscopies)) OR (Microscopies, Confocal)) OR (Laser Scanning Microscopy)) OR (Laser Scanning Microscopies)) OR (Microscopies, Laser Scanning)) OR (Microscopy, Laser Scanning)) OR (Scanning Microscopies, Laser)) OR (Scanning Microscopy, Laser)) OR (Microscopy, Confocal, Laser Scanning)) OR (Laser Scanning Confocal Microscopy)) OR (Confocal Laser Scanning Microscopy)) OR (Confocal Microscopy, Scanning Laser)) OR (Laser Microscopy)) OR (Laser Microscopies)) OR (Microscopies, Laser)) OR (Microscopy, Laser)
#14	(#12) OR (#13)
#15	Spectrum Analysis, Raman[MeSH Terms]
#16	((((((Raman Spectrum Analysis) OR (Raman Spectroscopy)) OR (Spectroscopy, Raman)) OR (Analysis, Raman Spectrum)) OR (Raman Optical Activity Spectroscopy)) OR (Raman Scattering)) OR (Scattering, Raman)
#17	(#15) OR (#16)
#18	Optical Imaging[MeSH Terms]
#19	((((((((Imaging, Optical) OR (Fluorescence Imaging)) OR (Imaging, Fluorescence)) OR (Fundus Autofluorescence Imaging)) OR (Autofluorescence Imaging, Fundus)) OR (Fundus Autofluorescence Imagings)) OR (Imaging, Fundus Autofluorescence)) OR (Autofluorescence Imaging)) OR (Imaging, Autofluorescence)
#20	(#18) OR (#19)
#21	Neural Networks, Computer[MeSH Terms]
#22	((((((((((((((((((((((((((Computer Neural Network) OR (Computer Neural Networks)) OR (Network, Computer Neural)) OR (Networks, Computer Neural)) OR (Neural Network, Computer)) OR (Models, Neural Network)) OR (Model, Neural Network)) OR (Network Model, Neural)) OR (Network Models, Neural)) OR (Neural Network Model)) OR (Neural Network Models)) OR (Computational Neural Networks)) OR (Computational Neural Network)) OR (Network, Computational Neural)) OR (Networks, Computational Neural)) OR (Neural Network, Computational)) OR (Neural Networks, Computational)) OR (Perceptrons)) OR (Perceptron)) OR (Connectionist Models)) OR (Connectionist Model)) OR (Model, Connectionist)) OR (Models, Connectionist)) OR (Neural Networks (Computer))) OR (Network, Neural (Computer))) OR (Networks, Neural (Computer))) OR (Neural Network (Computer))
#23	(#21) OR (#22)
#24	Diagnosis, Computer-Assisted[MeSH Terms]
#25	((((Diagnosis, Computer Assisted) OR (Computer-Assisted Diagnosis)) OR (Computer Assisted Diagnosis)) OR (Computer-Assisted Diagnoses)) OR (Diagnoses, Computer-Assisted)
#26	(#24) OR (#25)
#27	Artificial Intelligence[MeSH Terms]
#28	((((((((((((((((((Intelligence, Artificial) OR (Computational Intelligence)) OR (Intelligence, Computational)) OR (Machine Intelligence)) OR (Intelligence, Machine)) OR (Computer Reasoning)) OR (Reasoning, Computer)) OR (AI (Artificial Intelligence))) OR (Computer Vision Systems)) OR (Computer Vision System)) OR (System, Computer Vision)) OR (Systems, Computer Vision)) OR (Vision System, Computer)) OR (Vision Systems, Computer)) OR (Knowledge Acquisition (Computer))) OR (Acquisition, Knowledge (Computer))) OR (Knowledge Representation (Computer))) OR (Knowledge Representations (Computer))) OR (Representation, Knowledge (Computer))
#29	(#27) OR (#28)
#30	((((((((((((#3) AND (#4)) AND ((#3) AND (#7))) AND ((#3) AND (#8))) AND ((#3) AND (#9))) AND ((#3) AND (#10))) AND ((#3) AND (#11))) AND ((#3) AND (#14))) AND ((#3) AND (#17))) AND ((#3) AND (#20))) AND ((#3) AND (#23))) AND ((#3) AND (#26))) AND ((#3) AND (#29))

### 2.2 Inclusion criteria

1. The experimental group employed a novel endoscopic technique as a diagnostic measure for gastric *H. pylori* infection; 2. The gold standard for diagnosis included the Rapid Urease and Breath Test; 3. Diagnostic techniques comprised novel endoscopic methods and up to two diagnostic approaches; 4. The reported outcome indicators encompassed true positive (TP), true negative (TN), false positive (FP), false negative (FN), sensitivity (Se), specificity (Sp), positive predictive value (PPV), and negative predictive value (NPV). When TP, TN, FP, FN, NPV, or PPV were not reported, calculations were derived from known variables such as Se and Sp; 5. The study design adhered to a prospective or retrospective approach.

### 2.3 Exclusion criteria

1. Absence of well-defined inclusion and exclusion criteria; 2. Non-clinical investigations; 3. Excluded document types: guidelines, systematic reviews, meta-analyses, narrative reviews, letters, editorials, research protocols, case reports, short newsletters, etc.; 4. Incomplete research data, duplicated publications, etc. Studies meeting any of the exclusion criteria were excluded from the analysis.

### 2.4 Study selection

The literature was managed using EndNote X9.1 for screening and exclusion. Initially, the two researchers checked for duplications in literature titles, review papers, conference papers, protocols, and short communications. Subsequently, both researchers reviewed the literature’s abstracts to determine inclusion and exclusion criteria. The two researchers then comprehensively reviewed the remaining literature to finalize the inclusion scope. The researchers independently screened the literature throughout this process, and the results were compared. In discrepancies, a discussion ensued, and resolution was achieved with the involvement of a third researcher.

### 2.5 Data extraction

Data for inclusion in the study were recorded using a standardized, preselected nine-item data extraction form, categorized under the following headings: 1. Author, 2. Country, 3. Year of publication, 4. Mean age, 5. Total number of individuals and the distribution by sex, 6. Diagnostic methods, 7. Gold standard, 8. Sensitivity, 9. Specificity.

### 2.6 Literature quality evaluation

Two investigators independently conducted a quality assessment using the Quality Assessment of Diagnostic Accuracy Studies Tool (QUADAS-2), and the assessment results were cross-checked (Yang et al., 2021). Any disparities were deliberated upon and resolved by a third investigator. The evaluation scale encompassed the assessment of risk of bias and clinical applicability. The risk of bias evaluation included four sections: case selection, trial under consideration, gold standard, cash flow, and progress. All components underwent assessment for risk of bias, and the initial three components underwent assessment for clinical applicability. The risk of bias was categorized as “low,” “high,” or “uncertain.”

### 2.7 Data analysis

We conducted network meta-analysis aggregation and analysis employing Markov chain Monte Carlo simulation chains within a Bayesian framework, utilizing R software version 4.3.1, following the guidelines outlined in the PRISMA network meta-analysis manual (Moher et al., 2015). The resulting network diagram, generated by the R software, illustrates and describes various novel endoscopes. Each node on the network diagram signifies a distinct novel endoscopic technique, while the connecting lines represent direct head-to-head comparisons with the gold standard. The size of each node and the width of the connecting line are proportional to the number of studies conducted (Chaimani et al., 2013).

GeMTC parameters were configured with 50,000 simulation iterations, 20,000 rotation iterations, and four chains. Model convergence was assessed using the latent scale reduction factor (PSRF). Convergence is deemed satisfactory when the PSRF is close to 1, indicating reliable agreement of the homogeneity model for subsequent analysis.

### 2.8 Research, identification and selection

**Figure 1:**
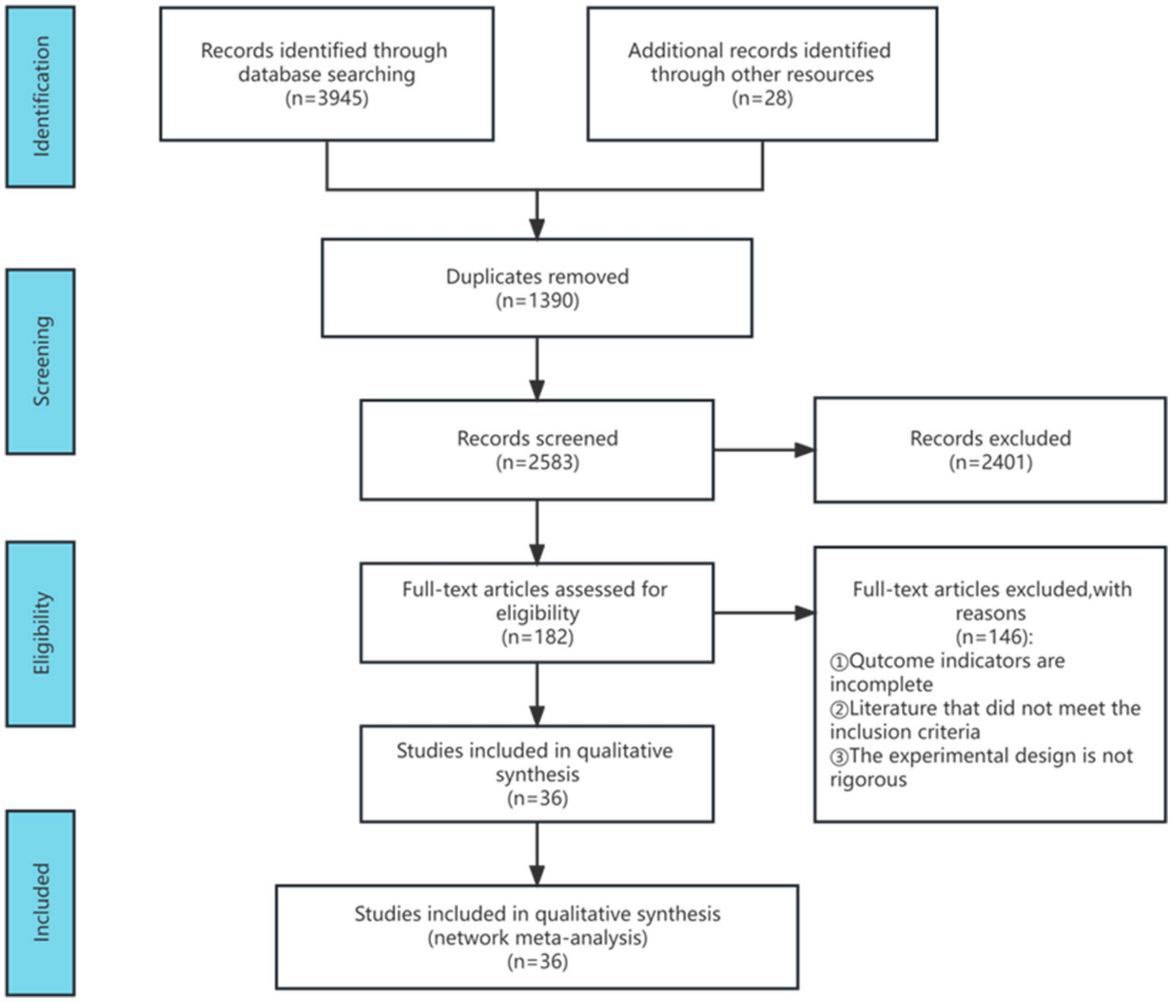
Flow diagram of literature selection.

Following the search strategy, a database inquiry yielded 3,973 articles. Post deduplication, 2,583 articles remained. Subsequently, 2,401 articles were excluded after reviewing titles and abstracts. The remaining 182 articles underwent thorough evaluation, leading to the removal of 146 due to incomplete outcome indicators, non-compliance with inclusion criteria, and insufficient experimental rigor. Ultimately, 36 articles were included in the meta-analysis, and Figure 1 illustrates the detailed literature screening process.

### 2.9 Characteristics of included studies

In total, 36 articles encompassing 7,230 patients diagnosed with gastric *H. pylori* infection through novel endoscopic techniques were included. Among them, 14 new endoscopic diagnostic methods were identified, comprising AI-BLI (1 study; Nakashima et al., 2018), AI-LCI (4 studies; Nakashima et al., 2018, 2020; Yasuda et al., 2020; Sun et al., 2023), AI-WLE (13 studies; Huang et al., 2004; Shichijo et al., 2017; Itoh et al., 2018; Nakashima et al., 2018; Shichijo et al., 2019; Zheng et al., 2019; Nakashima et al., 2020; Li et al., 2023; Seo et al., 2023; Zhang et al., 2023), BLI (1 study; Nishikawa et al., 2018), CLE (2 studies; Ji et al., 2010; Wang et al., 2010), LCI (15 studies; Dohi et al., 2016; Jiang et al., 2019; Sun et al., 2019; Wang et al., 2019; Lee et al., 2020; Ono et al., 2020; Xiu et al., 2021), M-BLI (1 study; Tahara et al., 2017), ME (3 studies; Gonen et al., 2009; Qi et al., 2013; Chen et al., 2018), M-I-SCAN (1 study; Qi et al., 2013), M-LCI (1 study; Chen et al., 2018), M-NBI (7 studies; Tahara et al., 2009; Liu et al., 2014; Yagi et al., 2014; Tahara et al., 2017, 2019; Cho et al., 2021), NBI (3 studies; Bansal et al., 2008; Özgür et al., 2015; Glover et al., 2021), OE-ME (1 study; Robles-Medranda et al., 2020), TXI-IEE (1 study; Kitagawa et al., 2023). Of these studies, 25 employed ①Rapid Urease Test, 33 used ②Urea Breath Test as the diagnostic gold standard, 39 relied on ③Gastromucosal Biopsy, 20 opted for ④Serological Examination, six utilized ⑤Fecal *H. pylori* Antigen Detection, and one employed ⑥*H. pylori* Culture is the gold standard for diagnosis. The studies originated from East Asia (49 studies), West Asia (2 studies), North America (1 study), South America (1 study), and Europe (1 study). Table 2 provides details on the characteristics of the included studies.

**Table 2:** Characteristics of the studies included in the meta-analysis.

Author	Country	Year	Age (Mean ± SD)	Total/Male/Female	Diagnostic method	Golden standard	Sensitivity	Specificity
Ono S	Japan	2019	62.4 ± 14.0	127/66/61	LCI	②④	0.844	0.889
Zhang M	China	2023	Internal test dataset: 46.46 ± 12.69 External test dataset: 48.73 ± 12.60	168/73/95 (Internal test dataset) 124/54/70 (External test dataset) 292/127/165 (Test dataset total)	AI-WLE	②③	0.929	0.893
Shichijo S	Japan	2019	NA	847/NA/NA	AI(CNN)-WLE	②④⑤	0.629	0.940
Shichijo S	Japan	2017	50.4 ± 11.2	397/171/226	AI(CNN)-WLE (First) AI(CNN)-WLE (Secondary)	②④⑤	0.819 0.889	0.834 0.874
Sun X	China	2023	Mean (Range): Patients with *H. pylori* infection (n = 10): 42.7 (18–68) Patients without *H. pylori* infection (n = 69): 44.5(18–67)	79/52/27	AI-LCI	①	0.681	0.679
Nakashima H	Japan	2018	AI training and test total 222 enrolled subjects: 55.1 ± 13.2	60/NA/NA (AI test)	AI(CNN)-WLE AI(CNN)-BLI AI(CNN)-LCI	④	0.667 0.967 0.967	0.600 0.867 0.833
Glover B	UK	2021	54.23 ± 16.80	153/66/87	NBI	①③	0.643	0.784
Itoh T	Japan	2018	NA	30/NA/NA	AI(CNN)-WLE	④	0.867	0.867
Li YD	China	2023	56.7 ± 12.4	191/101/90	AI(CNN)-WLE	②	0.833	0.858
Li YD	China	2023	NA	100/NA/NA (Videos from 100 cases in the database of ZJCH)	AI(CNN)-WLE	②	0.820	0.860
Kitagawa Y	Japan	2023	Median (IQR): 73(68–78)	60/41/19 (Observed five times) Total:300/205/95	TXI-IEE	②④	0.692	0.961
Nakashima H	Japan	2020	57.2 ± 12.9	120/NA/NA	AI(CAD)-LCI AI(CAD)-WLE	②④	0.625 0.600	0.925 0.862
Nishikawa Y	Japan	2018	65.6 ± 13.3	439/160/279	BLI	②③④	0.419	0.953
Wang P	China	2010	Median (Range): 49.8(19–67)	121/74/44	CLE	①②③	0.829	0.909
Wang L	China	2019	Median (Range): 48(26–82)	103/42/61	LCI(Corpus images) LCI(Antrum images)	①③	0.854 0.600	0.797 0.658
Tahara T	Japan	2019	Median (Range): 66(22–87)	163/NA/NA (Total 207 endoscopic examinations from 163 participants)	M-NBI(Reader A) M-NBI(Reader B)	②③④	0.969 0.928	0.936 0.936
Xiu JZ	China	2021	50.8 ± 13.4	392/155/237	LCI	①②③	0.919	0.911
Gonen C	Turkey	2009	48.6 ± 14.2	129/32/97	ME	①②③	0.939	0.903
Huang CR	Taiwan	2004	43.2 ± NA	104/NA/NA	AI(RFSNN)-WLE	③	0.854	0.909
Ji R	China	2009	Mean (Range): 47.2 (23–68)	83/47/36	CLE (Final diagnosis)	①②③	0.892	0.957
Bansal A	USA	2008	Mean (Range): 65(43–82)	47/46/1	NBI	③	0.750	0.880
Seo JY	Korea	2023	NA	702/NA/NA	AI(CNN)-WLE	①②③④⑥	0.819	0.930
Jiang ZX	China	2019	55.6 ± 22.8	358/140/218	Observer A: LCI(Score of 3.5) LCI(Score of 2.5) Observer B: LCI(Total score)	①②③	0.838 0.932 0.774	0.995 0.842 0.843
Liu H	China	2014	Mean (Range): 57.5 (33–82)	90/49/41	M-NBI	②③	0.750	0.791
Tahara T	Japan	2009	58.7 ± 13.6	106/64/42	M-NBI	③④	0.952	0.822
Zheng W	China	2019	48.6 ± 12.9	452/220/232	AI(CNN)-WLE (Multiple Gastric images) AI(CNN)-WLE (Single Gastric image)	②③	0.916 0.814	0.986 0.901
Qi QQ	China	2013	Mean (Range): 49.3(24–78)	84/47/37	ME M-I-SCAN	①②③	0.955 0.955	0.807 0.936
Robles-Medranda C	Ecuador	2020	46.3 ± 13.7	72/22/50	OE-ME	③⑤	0.914	0.784
Chen TH	Taiwan	2018	52.35 ± 12.90	122/70/52 (Final analysis of 111 patients)	M-LCI ME LCI	①②③	0.839 0.807 0.710	0.763 0.825 0.813
Dohi O	Japan	2016	*H. pylori*-positive (n = 30) Median: 29.0 *H. pylori*-negative (n = 30) Median: 65.5	60/37/23	LCI	①②③④	0.933	0.783
Tahara T	Japan	2017	Median (Range): 64 (22–87)	112/48/64	M-NBI	②③④	0.969	0.813
Tahara T	Japan	2017	Median (Range): 63 (24–86)	113/44/69	M-BLI	②③④	0.983	0.943
Yasuda T	Japan	2019	Median (IQR): 64 (26–88)	105/61/44	AI-LCI	②③④⑤	0.905	0.857
Yagi K	Japan	2013	NA	56/NA/NA	M-NBI	⑤	0.909	0.826
Cho JH	South Korea	2021	45.9 ± 14.6	254/119/135	M-NBI	①③	0.963	0.956
Özgür T	Turkey	2015	11.88 ± 4.55	165/68(Boys)/97(Girls)	NBI	③	0.929	0.624
Sun X	China	2019	Mean (Range): 47.20 (19–76)	127/66/61	LCI(Group A)	①③	0.906	0.790
Sun X	China	2019	Mean (Range): 49.66 (19–72)	126/68/58	LCI(Group B)	①③	0.906	0.877
Lee SP	Korea	2020	51.23 ± 15.01	100/52/48	LCI(ReaderA) LCI(ReaderB) LCI(ReaderC) LCI(ReaderD)	①③	0.676 0.595 0.351 0.676	0.937 0.937 0.905 0.873

①Rapid Urease Test. ②Urea Breath Test. ③Gastromucosal Biopsy. ④Serological Examination. ⑤Fecal *H. pylori* Antigen Detection. ⑥*H. pylori* Culture. AI(CAD), Artificial Intelligence-based Computer Aided Diagnosis. AI(CNN), Artificial Intelligence-based Convolutional Neural Networks. AI(RFSNN), Artificial Intelligence-based A Refined Feature Selection With Neural Network. BLI, Blue Laser Imaging Endoscopy. CLE, Confocal Laser Endomicroscopy. LCI, Linked Color Imaging Endoscopy. M-BLI, Magnifying Blue Laser Imaging Endoscopy. ME, Magnifying Endoscopy. M-I-SCAN, Magnifying Endoscopy Combined Newly Developed Image-Enhanced Endoscopy System With Special Functions. M-LCI, Magnifying Link Color Imaging Endoscopy. M-NBI, Magnifying Narrow-Band Imaging Endoscopy. NBI, Narrow-Band Imaging Endoscopy. OE-ME, Optically Enhanced Magnification Endoscopy. TXI-IEE, Texture And Color Enhancement Imaging And Image-Enhanced Endoscopy. WLE, White Light Imaging Endoscopy. NA, Not Assessable.

### 2.10 Quality assessment of included studies

**Figure 2:**
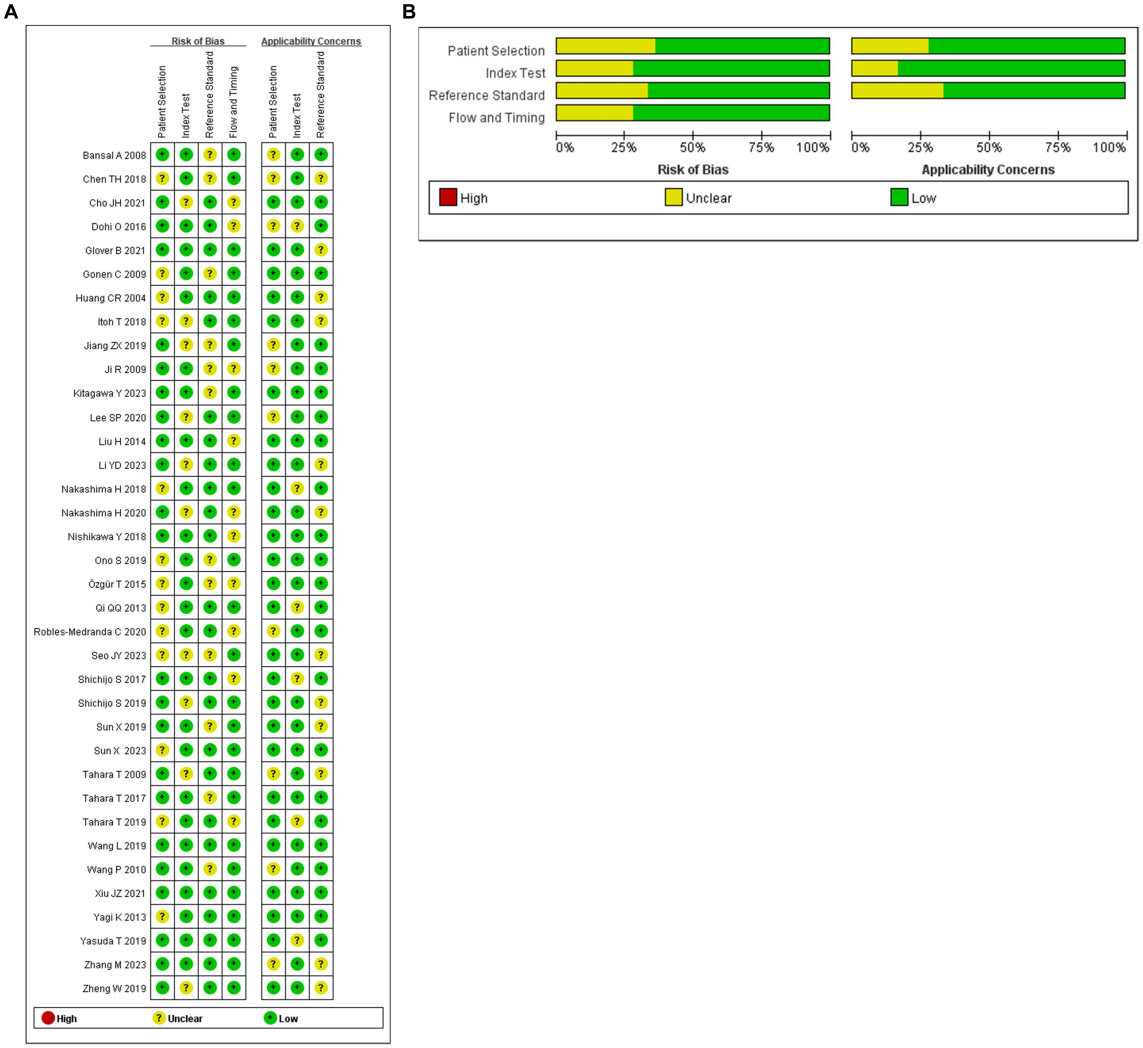
**(A)** Summary of risk of bias for each studies. **(B)** Proportion of risk of bias for all domains.

We utilized R software (version 4.3.1) to conduct a Bayesian network meta-analysis involving 36 articles, encompassing 54 observational studies. The quality, risk of bias, and applicability of these 36 articles were assessed using QUADAS-2. Overall, the articles demonstrated satisfactory quality, with 25 rated high quality and 11 as medium quality. Regarding personnel selection, 13 out of 36 articles had an unclear risk of bias, mandating informed consent from patients or their relatives before testing with new endoscopic techniques. Ten articles exhibited an unclear risk of bias in index detection, while 12 had a dark bias in reference standard assessment. The risk of bias in follow-up time was uncertain for 10 articles. Applicability considerations revealed no increased risk of bias in patient selection, reference standards, and index testing (refer to Figure 2 for details).

### 2.11 Network meta-analysis

**Figure 3:**
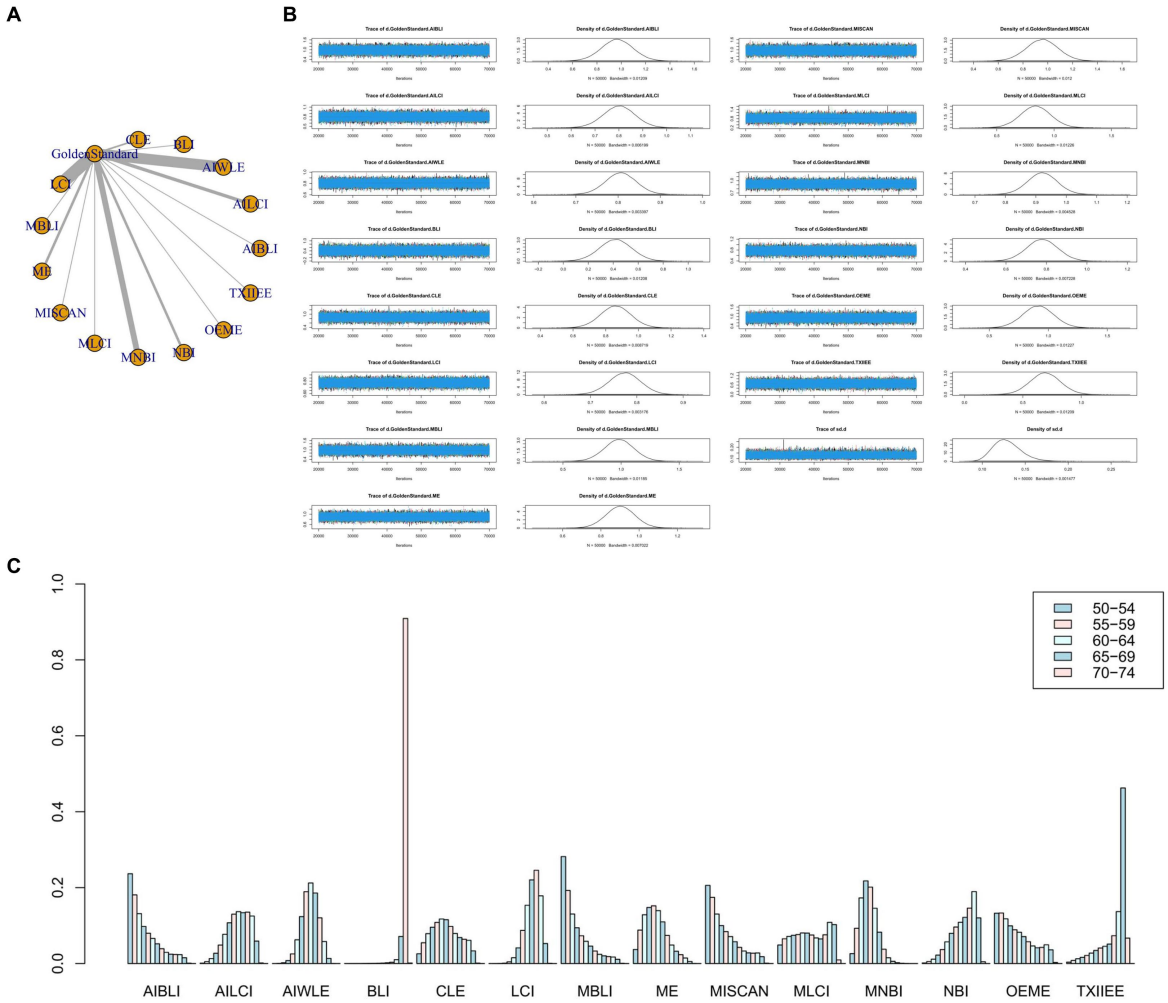
**(A)** Network meta-analysis figure for Sensitivity. **(B)** Convergence analysis for Sensitivity. **(C)** Ranking chart for Sensitivity.

**Figure 4:**
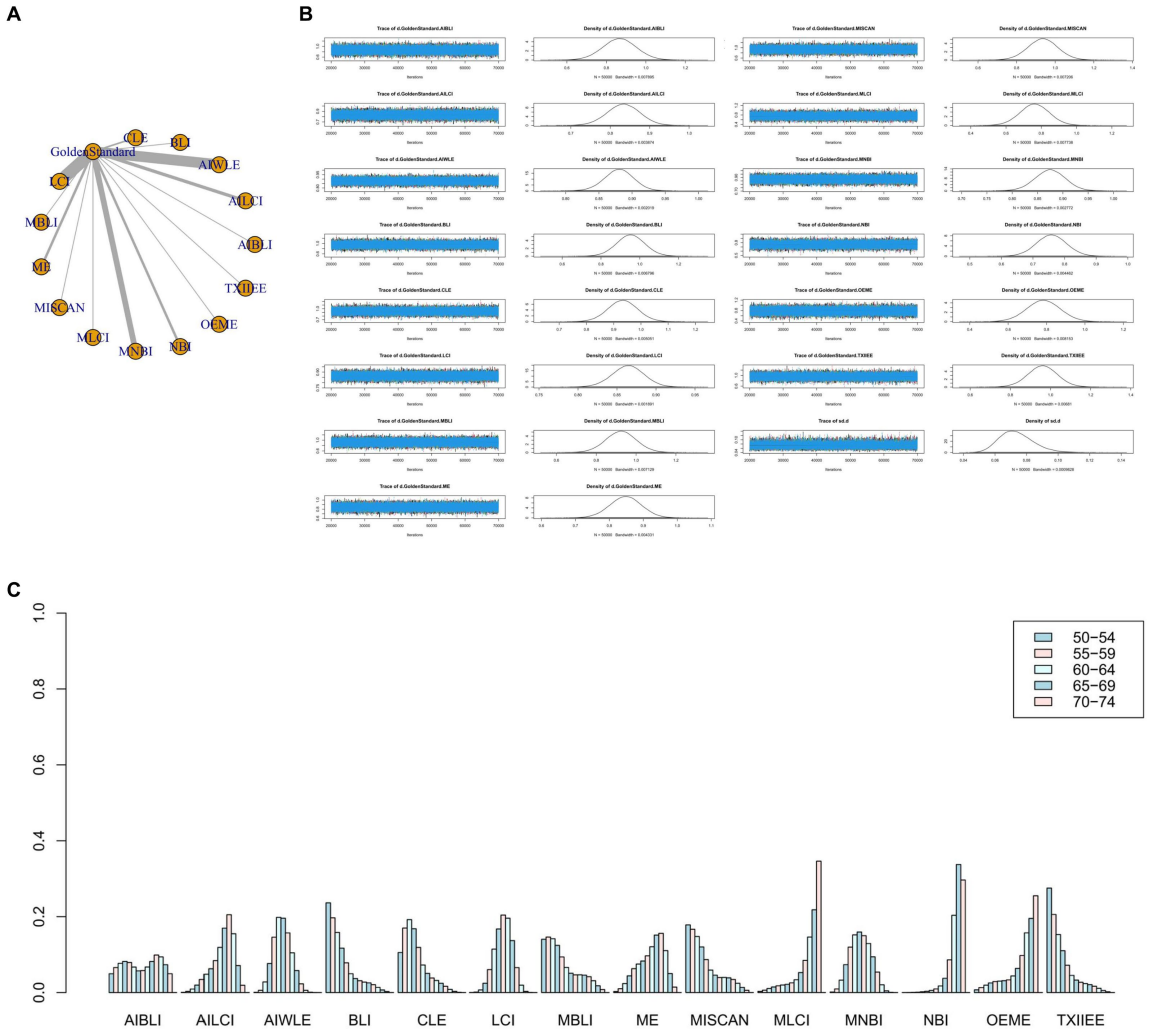
**(A)** Network meta-analysis figure for Specificity. **(B)** Convergence analysis for Specificity. **(C)** Ranking chart for Specificity.

**Figure 5:**
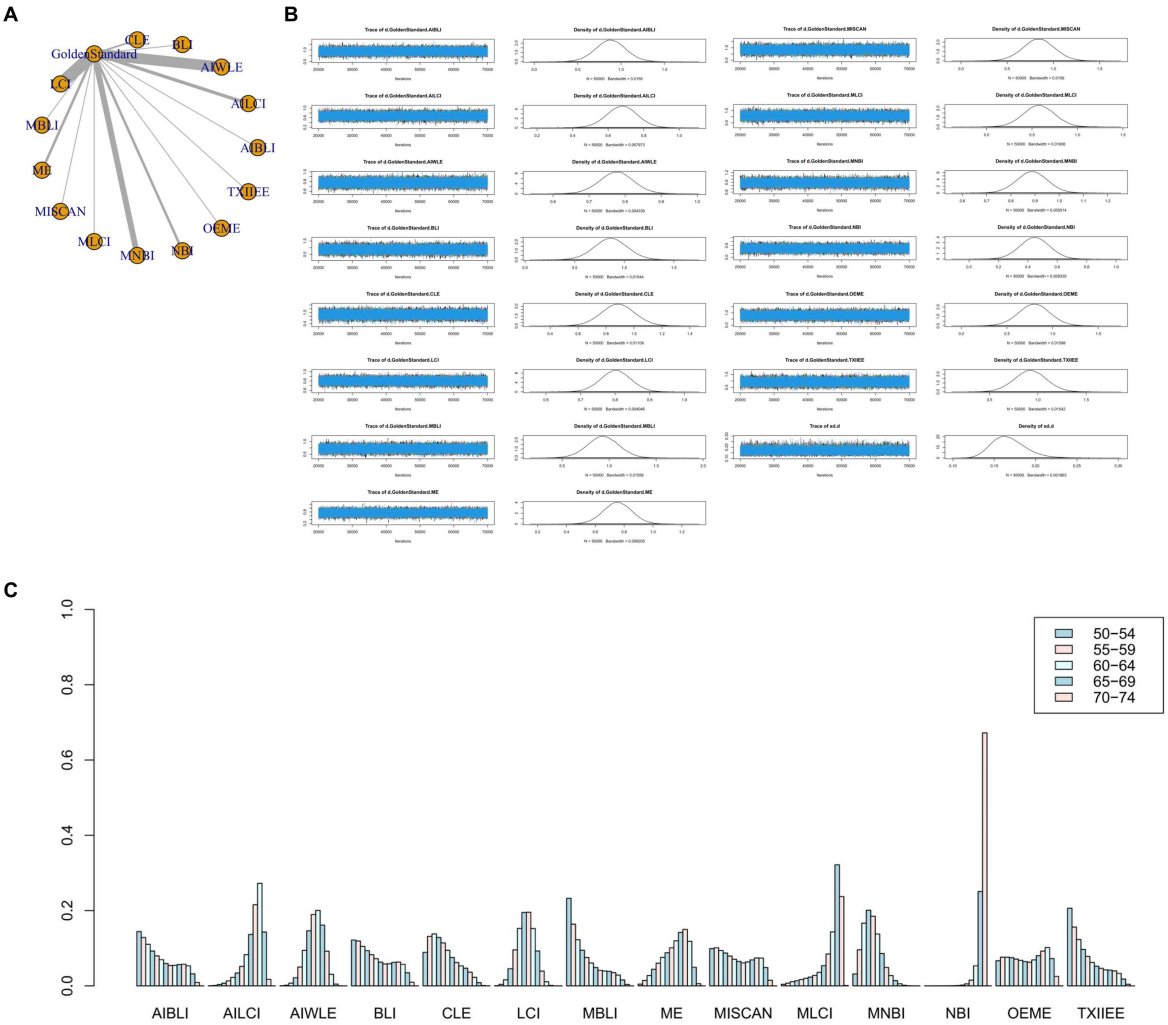
**(A)** Network meta-analysis figure for PPV. **(B)** Convergence analysis for PPV. **(C)** Ranking chart for PPV.

**Figure 6:**
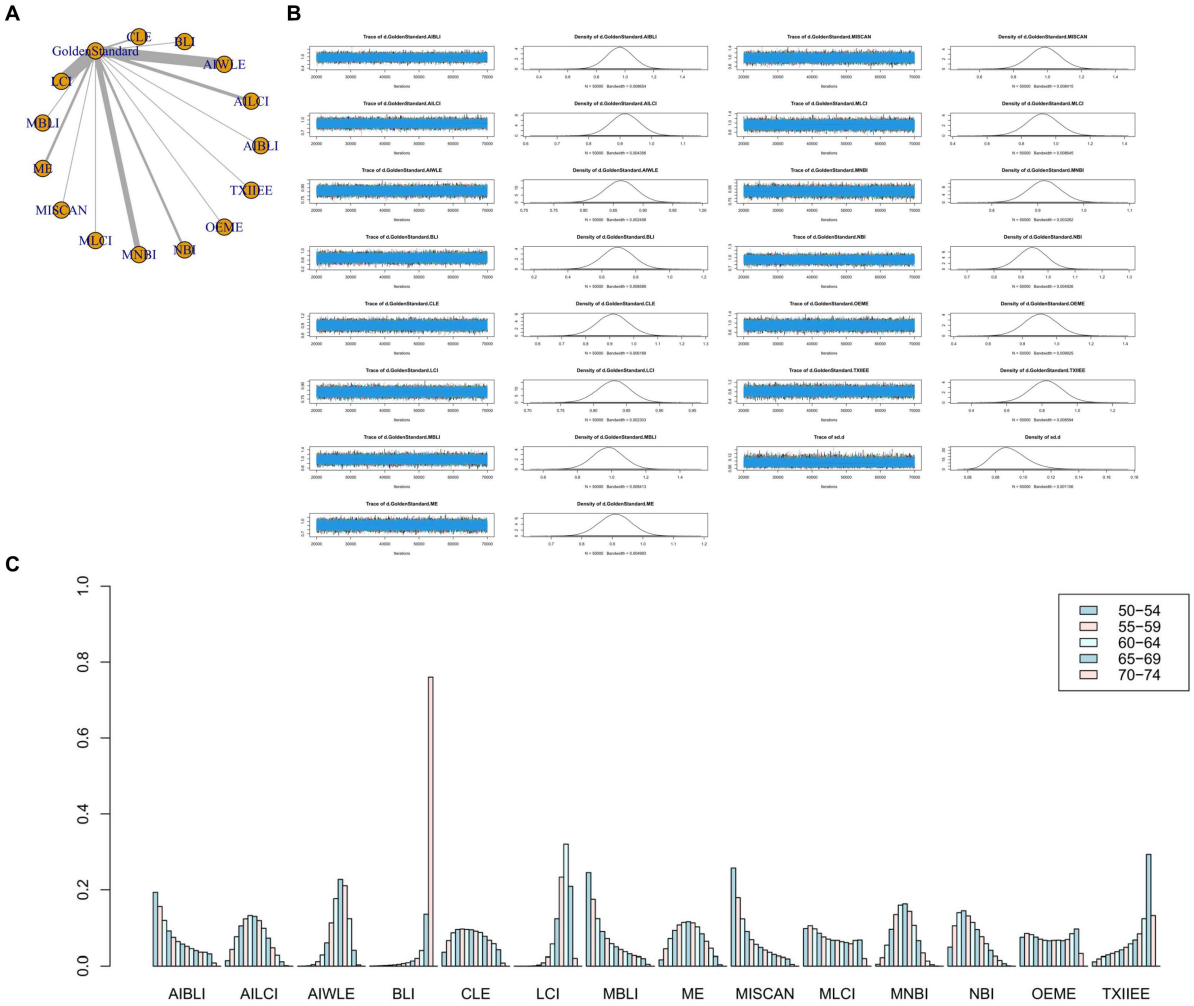
**(A)** Network meta-analysis figure for NPV. **(B)** Convergence analysis for NPV. **(C)** Ranking chart for NPV.

The full Network meta-analysis figure will be shown in Figures 3A, 4A, 5A, 6A.

#### 2.11.1 Sensitivity

In the results of the network meta-analysis, when compared to the gold standard detection, AI-BLI [MD = 0.966, 95%CI: (0.706, 1.227)], AI-LCI [MD = 0.801, 95%CI: (0.667, 0.935)], AI-WLE [MD = 0.806, 95%CI: (0.733, 0.880)], BLI [MD = 0.419, 95%CI: (0.158, 0.678)], CLE [MD = 0.861, 95%CI: (0.674, 1.048)], LCI [MD = 0.774, 95%CI: (0.705, 0.842)], M-BLI [MD = 0.984, 95% CI: (0.725, 1.242)], the ME [MD = 0.902, 95% CI: (0.749, 1.054)], M-I-SCAN [MD = 0.955, 95% CI: (0.695, 1.215)], M-LCI [MD = 0.839, 95% CI: (0.574, 1.104)], M-NBI [MD = 0.921, 95% CI: (0.824, 1.019)], NBI [MD = 0.779, 95% CI: (0.622, 0.935)], OE-ME [MD = 0.915, 95%CI: (0.650, 1.179)], TXI-IEE [MD = 0.691, 95%CI: (0.430, 0.954)], and the sensitivity differed from the gold standard, as indicated in Table 3. Convergence analysis confirmed the stability of the results, as depicted in Figure 3B. The bar chart illustrates the top five sensitivities in descending order: M-BLI (0.282), AI-BLI (0.237), M-I-SCAN (0.206), OE-ME (0.132), and M-LCI (0.049; Figure 3C). Table 3 presents a comparison between these two distinct detection measures.

**Table 3:** League table on sensitivity.

	AIBLI	AILCI	AIWLE	BLI	CLE	Goldenstandard	LCI	MBLI	ME	MISCAN	MLCI	MNBI	NBI	OEME	TXIIEE
AIBLI	AIBLI	−0.165 (−0.458, 0.126)	−0.160 (−0.430, 0.110)	−0.548 (−0.918, −0.179)	−0.106 (−0.427, 0.215)	−0.966 (−1.227, −0.706)	−0.193 (−0.462, 0.077)	0.0169 (−0.350, 0.384)	−0.065 (−0.367, 0.237)	−0.012 (−0.380, 0.355)	−0.128 (−0.498, 0.243)	−0.045 (−0.323, 0.233)	−0.188 (−0.492, 0.118)	−0.052 (−0.423, 0.320)	−0.275 (−0.644, 0.095)
AILCI	0.165 (−0.126, 0.458)	AILCI	0.005 (−0.146, 0.158)	−0.383 (−0.675, −0.090)	0.060 (−0.169, 0.290)	−0.801 (−0.935, −0.667)	−0.027 (−0.177, 0.123)	0.182 (−0.108, 0.475)	0.101 (−0.102, 0.304)	0.154 (−0.136, 0.447)	0.038 (−0.259, 0.336)	0.120 (−0.045, 0.286)	−0.022 (−0.227, 0.183)	0.114 (−0.183, 0.409)	−0.110 (−0.402, 0.185)
AIWLE	0.160 (−0.110, 0.430)	−0.005 (−0.158, 0.146)	AIWLE	−0.388 (−0.659, −0.119)	0.054 (−0.147, 0.256)	−0.806 (−0.880, −0.733)	−0.032 (−0.133, 0.067)	0.177 (−0.091, 0.446)	0.095 (−0.073, 0.263)	0.148 (−0.122, 0.417)	0.032 (−0.242, 0.307)	0.115 (−0.007, 0.237)	−0.028 (−0.201, 0.144)	0.108 (−0.166, 0.383)	−0.115 (−0.387, 0.158)
BLI	0.548 (0.179, 0.918)	0.383 (0.090, 0.675)	0.388 (0.119, 0.659)	BLI	0.442 (0.123, 0.763)	−0.419 (−0.678, −0.158)	0.355 (0.087, 0.625)	0.565 (0.197, 0.933)	0.483 (0.182, 0.786)	0.536 (0.169, 0.904)	0.420 (0.049, 0.792)	0.502 (0.225, 0.781)	0.360 (0.056, 0.665)	0.497 (0.124, 0.869)	0.273 (−0.096, 0.643)
CLE	0.106 (−0.215, 0.427)	−0.060 (−0.290, 0.169)	−0.054 (−0.256, 0.147)	−0.442 (−0.763, −0.123)	CLE	−0.861 (−1.048, −0.674)	−0.087 (−0.288, 0.112)	0.123 (−0.196, 0.442)	0.041 (−0.200, 0.282)	0.094 (−0.226, 0.415)	−0.022 (−0.345, 0.303)	0.061 (−0.151, 0.271)	−0.082 (−0.326, 0.161)	0.054 (−0.269, 0.379)	−0.169 (−0.489, 0.151)
Golden standard	0.966 (0.706, 1.227)	0.801 (0.667, 0.935)	0.806 (0.733, 0.880)	0.419 (0.158, 0.678)	0.861 (0.674, 1.048)	Goldenstandard	0.774 (0.705, 0.842)	0.984 (0.725, 1.242)	0.902 (0.749, 1.054)	0.955 (0.695, 1.215)	0.839 (0.574, 1.104)	0.921 (0.824, 1.019)	0.779 (0.622, 0.935)	0.915 (0.650, 1.179)	0.691 (0.430, 0.954)
LCI	0.193 (−0.077, 0.462)	0.027 (−0.123, 0.177)	0.032 (−0.067, 0.133)	−0.355 (−0.625, −0.087)	0.087 (−0.112, 0.288)	−0.774 (−0.842, −0.705)	LCI	0.209 (−0.057, 0.478)	0.128 (−0.038, 0.294)	0.181 (−0.087, 0.451)	0.064 (−0.209, 0.339)	0.147 (0.028, 0.267)	0.005 (−0.166, 0.176)	0.141 (−0.133, 0.415)	−0.083 (−0.352, 0.188)
MBLI	−0.017 (−0.384, 0.350)	−0.182 (−0.475, 0.108)	−0.177 (−0.446, 0.091)	−0.565 (−0.933, −0.197)	−0.123 (−0.442, 0.196)	−0.984 (−1.242, −0.725)	−0.209 (−0.478, 0.057)	MBLI	−0.082 (−0.381, 0.218)	−0.029 (−0.395, 0.338)	−0.145 (−0.516, 0.227)	−0.063 (−0.338, 0.214)	−0.205 (−0.506, 0.098)	−0.069 (−0.438, 0.300)	−0.292 (−0.659, 0.075)
ME	0.065 (−0.237, 0.367)	−0.101 (−0.304, 0.102)	−0.095 (−0.263, 0.073)	−0.483 (−0.786, −0.182)	−0.041 (−0.282, 0.200)	−0.902 (−1.054, −0.749)	−0.128 (−0.294, 0.038)	0.082 (−0.218, 0.381)	ME	0.053 (−0.248, 0.354)	−0.063 (−0.368, 0.243)	0.020 (−0.161, 0.199)	−0.123 (−0.341, 0.096)	0.013 (−0.292, 0.319)	−0.210 (−0.512, 0.092)
MISCAN	0.0116 (−0.355, 0.380)	−0.154 (−0.447, 0.136)	−0.148 (−0.417, 0.122)	−0.536 (−0.904, −0.169)	−0.094 (−0.415, 0.226)	−0.955 (−1.215, −0.695)	−0.181 (−0.451, 0.087)	0.029 (−0.338, 0.395)	−0.053 (−0.354, 0.248)	MISCAN	−0.116 (−0.487, 0.257)	−0.034 (−0.311, 0.244)	−0.176 (−0.480, 0.127)	−0.040 (−0.411, 0.331)	−0.264 (−0.631, 0.105)
MLCI	0.128 (−0.243, 0.498)	−0.038 (−0.336, 0.259)	−0.032 (−0.307, 0.242)	−0.420 (−0.792, −0.049)	0.022 (−0.303, 0.345)	−0.839 (−1.104, −0.574)	−0.064 (−0.339, 0.209)	0.145 (−0.227, 0.516)	0.063 (−0.243, 0.368)	0.116 (−0.257, 0.487)	MLCI	0.083 (−0.201, 0.365)	−0.060 (−0.368, 0.246)	0.076 (−0.300, 0.450)	−0.147 (−0.522, 0.225)
MNBI	0.045 (−0.233, 0.323)	−0.120 (−0.286, 0.045)	−0.115 (−0.237, 0.007)	−0.502 (−0.781, −0.225)	−0.061 (−0.271, 0.151)	−0.921 (−1.019, −0.824)	−0.147 (−0.267, −0.028)	0.063 (−0.214, 0.338)	−0.020 (−0.199, 0.161)	0.034 (−0.244, 0.311)	−0.083 (−0.365, 0.201)	MNBI	−0.143 (−0.327, 0.042)	−0.006 (−0.289, 0.275)	−0.230 (−0.509, 0.050)
NBI	0.188 (−0.118, 0.492)	0.022 (−0.183, 0.227)	0.028 (−0.144, 0.201)	−0.360 (−0.665, −0.056)	0.082 (−0.161, 0.326)	−0.779 (−0.935, −0.622)	−0.005 (−0.176, 0.166)	0.205 (−0.098, 0.506)	0.123 (−0.096, 0.341)	0.176 (−0.127, 0.480)	0.060 (−0.246, 0.368)	0.143 (−0.042, 0.327)	NBI	0.136 (−0.171, 0.444)	−0.088 (−0.390, 0.218)
OEME	0.052 (−0.320, 0.423)	−0.114 (−0.409, 0.183)	−0.108 (−0.383, 0.166)	−0.497 (−0.869, −0.124)	−0.054 (−0.379, 0.269)	−0.915 (−1.179, −0.650)	−0.141 (−0.415, 0.133)	0.069 (−0.300, 0.438)	−0.0130 (−0.319, 0.292)	0.040 (−0.331, 0.411)	−0.076 (−0.451, 0.300)	0.006 (−0.275, 0.289)	−0.136 (−0.444, 0.171)	OEME	−0.223 (−0.597, 0.150)
TXIIEE	0.275 (−0.095, 0.644)	0.110 (−0.185, 0.402)	0.115 (−0.158, 0.387)	−0.273 (−0.643, 0.096)	0.169 (−0.151, 0.489)	−0.691 (−0.954, −0.430)	0.083 (−0.188, 0.352)	0.292 (−0.075, 0.659)	0.210 (−0.092, 0.512)	0.264 (−0.105, 0.631)	0.147 (−0.225, 0.522)	0.230 (−0.050, 0.509)	0.088 (−0.218, 0.390)	0.223 (−0.150, 0.597)	TXIIEE

#### 2.11.2 Specificity

The network meta-analysis results indicated differences in specificity compared to the gold standard for various endoscopic techniques: AI-BLI [MD = 0.867, 95%CI: (0.697, 1.037)], AI-LCI [MD = 0.834, 95%CI: (0.750, 0.916)], AI-WLE [MD = 0.880, 95%CI: (0.836, 0.924)], BLI [MD = 0.953, 95%CI: (0.805, 1.102)], CLE [MD = 0.933, 95%CI: (0.824, 1.042)], LCI [MD = 0.864, 95%CI: (0.822, 0.905)], M-BLI [MD = 0.924, 95% CI: (0.770, 1.079)], ME [MD = 0.848, 95% CI: (0.754, 0.941)], M-I-SCAN [MD = 0.936, 95% CI: (0.780, 1.092)], M-LCI [MD = 0.762, 95% CI: (0.595, 0.930)], M-NBI [MD = 0.875, 95% CI: (0.814, 0.934)], NBI [MD = 0.758, 95% CI: (0.663, 0.855)], OE-ME [MD = 0.784, 95%CI: (0.609, 0.959)], TXI-IEE [MD = 0.961, 95%CI: (0.812, 1.110)] (Table 4). Convergence analysis demonstrated the stability of the results, as illustrated in Figure 4B. The ranked bar chart revealed the top five specificities in descending order: TXI-IEE (0.275), BLI (0.236), M-I-SCAN (0.178), M-BLI (0.140), and CLE (0.105; Figure 4C). Table 4 provides a comparison between these two distinct measures of detection.

**Table 4:** League table on specificity.

	AIBLI	AILCI	AIWLE	BLI	CLE	Goldenstandard	LCI	MBLI	ME	MISCAN	MLCI	MNBI	NBI	OEME	TXIIEE
AIBLI	AIBLI	−0.033 (−0.223, 0.156)	0.013 (−0.162, 0.189)	0.087 (−0.139, 0.311)	0.066 (−0.136, 0.268)	−0.8671 (−1.037, −0.697)	−0.003 (−0.178, 0.172)	0.058 (−0.172, 0.288)	−0.019 (−0.214, 0.174)	0.069 (−0.162, 0.299)	−0.104 (−0.343, 0.134)	0.008 (−0.173, 0.187)	−0.108 (−0.303, 0.088)	−0.083 (−0.327, 0.161)	0.094 (−0.132, 0.320)
AILCI	0.033 (−0.156, 0.223)	AILCI	0.047 (−0.047, 0.141)	0.119 (−0.050, 0.291)	0.099 (−0.037, 0.237)	−0.834 (−0.916, −0.750)	0.0311 (−0.062, 0.124)	0.091 (−0.084, 0.268)	0.014 (−0.110, 0.140)	0.102 (−0.074, 0.280)	−0.072 (−0.257, 0.116)	0.041 (−0.061, 0.144)	−0.076 (−0.201, 0.053)	−0.050 (−0.243, 0.145)	0.127 (−0.042, 0.299)
AIWLE	−0.013 (−0.189, 0.162)	−0.047 (−0.141, 0.047)	AIWLE	0.072 (−0.081, 0.228)	0.053 (−0.065, 0.171)	−0.880 (−0.924, −0.836)	−0.016 (−0.076, 0.044)	0.044 (−0.116, 0.206)	−0.032 (−0.136, 0.071)	0.055 (−0.106, 0.218)	−0.118 (−0.290, 0.055)	−0.006 (−0.080, 0.069)	−0.122 (−0.227, −0.015)	−0.097 (−0.277, 0.085)	0.080 (−0.074, 0.236)
BLI	−0.086 (−0.311, 0.139)	−0.119 (−0.291, 0.050)	−0.072 (−0.228, 0.081)	BLI	−0.020 (−0.204, 0.165)	−0.953 (−1.102, −0.805)	−0.089 (−0.244, 0.064)	−0.029 (−0.243, 0.185)	−0.105 (−0.281, 0.069)	−0.017 (−0.232, 0.198)	−0.190 (−0.414, 0.033)	−0.078 (−0.239, 0.081)	−0.195 (−0.371, −0.018)	−0.169 (−0.397, 0.060)	0.008 (−0.202, 0.217)
CLE	−0.066 (−0.268, 0.136)	−0.099 (−0.237, 0.037)	−0.053 (−0.171, 0.065)	0.020 (−0.165, 0.204)	CLE	−0.933 (−1.042, −0.824)	−0.069 (−0.186, 0.047)	−0.009 (−0.197, 0.181)	−0.085 (−0.229, 0.058)	0.003 (−0.188, 0.193)	−0.171 (−0.370, 0.029)	−0.058 (−0.183, 0.065)	−0.175 (−0.319, −0.029)	−0.150 (−0.355, 0.058)	0.028 (−0.156, 0.212)
Golden standard	0.867 (0.697, 1.037)	0.834 (0.750, 0.916)	0.880 (0.836, 0.924)	0.953 (0.805, 1.102)	0.933 (0.824, 1.042)	GoldenStandard	0.864 (0.822, 0.905)	0.924 (0.770, 1.079)	0.848 (0.754, 0.941)	0.936 (0.780, 1.092)	0.762 (0.595, 0.930)	0.875 (0.814, 0.934)	0.758 (0.663, 0.855)	0.784 (0.609, 0.959)	0.961 (0.812, 1.110)
LCI	0.003 (−0.172, 0.178)	−0.031 (−0.124, 0.062)	0.016 (−0.044, 0.076)	0.089 (−0.064, 0.244)	0.069 (−0.047, 0.186)	−0.864 (−0.905, −0.822)	LCI	0.060 (−0.098, 0.221)	−0.016 (−0.118, 0.086)	0.072 (−0.090, 0.233)	−0.102 (−0.274, 0.071)	0.011 (−0.062, 0.083)	−0.106 (−0.209, 0.000)	−0.080 (−0.260, 0.100)	0.097 (−0.056, 0.252)
MBLI	−0.058 (−0.288, 0.172)	−0.091 (−0.268, 0.084)	−0.044 (−0.206, 0.116)	0.029 (−0.185, 0.243)	0.009 (−0.181, 0.197)	−0.924 (−1.079, −0.770)	−0.060 (−0.221, 0.098)	MBLI	−0.076 (−0.259, 0.104)	0.012 (−0.208, 0.231)	−0.162 (−0.389, 0.065)	−0.049 (−0.216, 0.115)	−0.166 (−0.348, 0.015)	−0.140 (−0.375, 0.093)	0.037 (−0.178, 0.250)
ME	0.019 (−0.174, 0.214)	−0.014 (−0.140, 0.110)	0.032 (−0.071, 0.136)	0.105 (−0.069, 0.281)	0.085 (−0.058, 0.229)	−0.848 (−0.941, −0.754)	0.016 (−0.086, 0.118)	0.076 (−0.104, 0.259)	ME	0.088 (−0.094, 0.271)	−0.086 (−0.276, 0.107)	0.027 (−0.084, 0.138)	−0.090 (−0.222, 0.045)	−0.064 (−0.263, 0.135)	0.113 (−0.062, 0.289)
MISCAN	−0.069 (−0.300, 0.162)	−0.102 (−0.280, 0.074)	−0.055 (−0.218, 0.106)	0.017 (−0.198, 0.232)	−0.003 (−0.193, 0.188)	−0.936 (−1.092, −0.780)	−0.072 (−0.233, 0.090)	−0.012 (−0.231, 0.208)	−0.088 (−0.271, 0.094)	MISCAN	−0.173 (−0.402, 0.055)	−0.061 (−0.229, 0.106)	−0.178 (−0.359, 0.006)	−0.152 (−0.387, 0.082)	0.025 (−0.191, 0.240)
MLCI	0.104 (−0.134, 0.343)	0.072 (−0.116, 0.257)	0.118 (−0.055, 0.290)	0.190 (−0.033, 0.414)	0.171 (−0.029, 0.370)	−0.762 (−0.930, −0.595)	0.102 (−0.071, 0.274)	0.162 (−0.065, 0.389)	0.086 (−0.107, 0.276)	0.173 (−0.055, 0.402)	MLCI	0.113 (−0.066, 0.290)	−0.004 (−0.196, 0.189)	0.021 (−0.220, 0.264)	0.198 (−0.024, 0.423)
MNBI	−0.008 (−0.187, 0.173)	−0.041 (−0.144, 0.061)	0.006 (−0.069, 0.080)	0.078 (−0.081, 0.239)	0.058 (−0.065, 0.183)	−0.875 (−0.934, −0.814)	−0.011 (−0.083, 0.062)	0.049 (−0.115, 0.216)	−0.027 (−0.138, 0.084)	0.061 (−0.106, 0.229)	−0.113 (−0.290, 0.066)	MNBI	−0.117 (−0.229, −0.003)	−0.091 (−0.275, 0.095)	0.086 (−0.073, 0.247)
NBI	0.108 (−0.088, 0.303)	0.076 (−0.053, 0.201)	0.122 (0.015, 0.227)	0.195 (0.018, 0.371)	0.175 (0.029, 0.319)	−0.758 (−0.855, −0.663)	0.106 (0.000, 0.209)	0.166 (−0.015, 0.348)	0.090 (−0.045, 0.222)	0.178 (−0.006, 0.359)	0.004 (−0.189, 0.196)	0.117 (0.003, 0.229)	NBI	0.026 (−0.175,0.225)	0.203 (0.025, 0.379)
OEME	0.083 (−0.161, 0.327)	0.050 (−0.145, 0.243)	0.097 (−0.085, 0.277)	0.169 (−0.060, 0.397)	0.150 (−0.058, 0.356)	−0.784 (−0.959, −0.609)	0.080 (−0.010, 0.260)	0.140 (−0.093, 0.375)	0.064 (−0.135, 0.263)	0.152 (−0.082, 0.387)	−0.021 (−0.264, 0.220)	0.091 (−0.095, 0.275)	−0.026 (−0.225, 0.175)	OEME	0.177 (−0.053, 0.407)
TXIIEE	−0.094 (−0.320, 0.132)	−0.127 (−0.299, 0.042)	−0.080 (−0.236, 0.074)	−0.008 (−0.217, 0.202)	−0.028 (−0.212, 0.156)	−0.961 (−1.110, −0.812)	−0.097 (−0.252, 0.056)	−0.037 (−0.250, 0.178)	−0.113 (−0.289, 0.062)	−0.025 (−0.240, 0.191)	−0.198 (−0.423, 0.024)	−0.086 (−0.247, 0.073)	−0.203 (−0.379, −0.025)	−0.177 (−0.407, 0.053)	TXIIEE

#### 2.11.3 Positive predictive value

Network meta-analysis results revealed differences from the gold standard in terms of positive predictive value for various endoscopic techniques: AI-BLI [MD = 0.879, 95%CI: (0.536, 1.224)], AI-LCI [MD = 0.678, 95%CI: (0.507, 0.849)], AI-WLE [MD = 0.776, 95% CI: (0.682, 0.870)], BLI [MD = 0.865, 95% CI: (0.531, 1.201)], CLE [MD = 0.886, 95%CI: (0.647, 1.126)], LCI [MD = 0.802, 95%CI: (0.714, 0.889)], M-BLI [MD = 0.935, 95% CI: (0.597, 1.270)], ME [MD = 0.754, 95% CI: (0.555, 0.951)], M-I-SCAN [MD = 0.840, 95% CI: (0.497, 1.183)], M-LCI [MD = 0.577, 95% CI: (0.233, 0.923)], M-NBI [MD = 0.888, 95% CI: (0.760, 1.015)], NBI [MD = 0.447, 95% CI: (0.246, 0.647)], OE-ME [MD = 0.799, 95%CI: (0.454, 1.146)], TXI-IEE [MD = 0.922, 95%CI: (0.588, 1.256)] (Table 5). Convergence analysis demonstrated the stability of the results, as illustrated in Figure 5B. The ranked histogram revealed the top five positive predictive values in descending order: M-BLI (0.232), TXI-IEE(0.206), AI-BLI(0.144), BLI(0.122), and M-I-SCAN(0.099; Figure 5C). Table 5 provides a comparison between these two distinct measures of detection.

**Table 5:** League table on PPV.

	AIBLI	AILCI	AIWLE	BLI	CLE	Golden standard	LCI	MBLI	ME	MISCAN	MLCI	MNBI	NBI	OEME	TXIIEE
AIBLI	AIBLI	−0.201 (−0.586, 0.182)	−0.103 (−0.461, 0.253)	−0.014 (−0.493, 0.466)	0.007 (−0.413, 0.424)	−0.879 (−1.224, −0.536)	−0.078 (−0.433, 0.277)	0.055 (−0.422, 0.535)	−0.126 (−0.522, 0.270)	−0.039 (−0.524, 0.441)	−0.303 (−0.788, 0.186)	0.009 (−0.359, 0.375)	−0.432 (−0.831, −0.035)	−0.080 (−0.566, 0.409)	0.043 (−0.437, 0.521)
AILCI	0.201 (−0.182, 0.586)	AILCI	0.098 (−0.098, 0.294)	0.187 (−0.189, 0.566)	0.208 (−0.087, 0.503)	−0.678 (−0.849, −0.507)	0.124 (−0.069, 0.315)	0.257 (−0.121, 0.634)	0.076 (−0.187, 0.337)	0.162 (−0.220, 0.545)	−0.102 (−0.485, 0.285)	0.210 (−0.004, 0.423)	−0.231 (−0.494, 0.034)	0.122 (−0.263, 0.507)	0.244 (−0.131, 0.620)
AIWLE	0.103 (−0.253, 0.461)	−0.098 (−0.294, 0.098)	AIWLE	0.089 (−0.258, 0.438)	0.110 (−0.147, 0.367)	−0.776 (−0.870, −0.682)	0.026 (−0.102, 0.154)	0.159 (−0.190, 0.508)	−0.022 (−0.242, 0.196)	0.064 (−0.292, 0.418)	−0.200 (−0.555, 0.158)	0.112 (−0.047, 0.270)	−0.329 (−0.550, −0.108)	0.024 (−0.334, 0.381)	0.146 (−0.201, 0.494)
BLI	0.014 (−0.466, 0.493)	−0.187 (−0.566, 0.189)	−0.089 (−0.438, 0.258)	BLI	0.021 (−0.392, 0.432)	−0.865 (−1.201, −0.531)	−0.063 (−0.412, 0.281)	0.070 (−0.404, 0.543)	−0.111 (−0.502, 0.276)	−0.025 (−0.505, 0.451)	−0.288 (−0.768, 0.193)	0.023 (−0.337, 0.382)	−0.418 (−0.809, −0.027)	−0.066 (−0.549, 0.416)	0.057 (−0.418, 0.530)
CLE	−0.007 (−0.424, 0.413)	−0.208 (−0.503, 0.087)	−0.110 (−0.367, 0.147)	−0.021 (−0.432, 0.392)	CLE	−0.886 (−1.126, −0.647)	−0.084 (−0.340, 0.171)	0.049 (−0.365, 0.461)	−0.132 (−0.442, 0.177)	−0.046 (−0.465, 0.373)	−0.309 (−0.729, 0.111)	0.002 (−0.270, 0.273)	−0.439 (−0.752, −0.126)	−0.086 (−0.508, 0.333)	0.036 (−0.375, 0.448)
Golden standard	0.879 (0.536, 1.224)	0.678 (0.507, 0.849)	0.776 (0.682, 0.870)	0.865 (0.531, 1.201)	0.886 (0.647, 1.126)	GoldenStandard	0.802 (0.714, 0.889)	0.935 (0.597, 1.270)	0.754 (0.555, 0.951)	0.840 (0.497, 1.183)	0.577 (0.233, 0.923)	0.888 (0.760, 1.015)	0.447 (0.246, 0.647)	0.799 (0.454, 1.146)	0.922 (0.588, 1.256)
LCI	0.078 (−0.277, 0.433)	−0.124 (−0.315, 0.069)	−0.026 (−0.154, 0.102)	0.063 (−0.281, 0.412)	0.084 (−0.171, 0.340)	−0.802 (−0.889, −0.714)	LCI	0.134 (−0.215, 0.480)	−0.048 (−0.265, 0.167)	0.038 (−0.315, 0.390)	−0.225 (−0.580, 0.131)	0.086 (−0.069, 0.241)	−0.355 (−0.574, −0.136)	−0.002 (−0.359, 0.354)	0.120 (−0.225, 0.468)
MBLI	−0.055 (−0.535, 0.422)	−0.257 (−0.634, 0.121)	−0.159 (−0.508, 0.190)	−0.070 (−0.543, 0.404)	−0.049 (−0.461, 0.365)	−0.935 (−1.270, −0.597)	−0.134 (−0.480, 0.215)	MBLI	−0.181 (−0.572, 0.210)	−0.096 (−0.574, 0.384)	−0.359 (−0.840, 0.125)	−0.047 (−0.4057, 0.3147)	−0.489 (−0.8807, −0.097)	−0.136 (−0.6177, 0.347)	−0.013 (−0.488, 0.461)
ME	0.126 (−0.270, 0.522)	−0.076 (−0.337, 0.187)	0.022 (−0.196, 0.242)	0.111 (−0.276, 0.502)	0.132 (−0.177, 0.442)	−0.754 (−0.951, −0.555)	0.048 (−0.167, 0.265)	0.181 (−0.210, 0.572)	ME	0.085 (−0.309, 0.482)	−0.178 (−0.575, 0.222)	0.134 (−0.102, 0.370)	−0.307 (−0.589, −0.024)	0.046 (−0.352, 0.445)	0.168 (−0.218, 0.558)
MISCAN	0.039 (−0.441, 0.524)	−0.162 (−0.545, 0.220)	−0.064 (−0.418, 0.292)	0.025 (−0.451, 0.505)	0.046 (−0.373, 0.465)	−0.840 (−1.183, −0.497)	−0.038 (−0.390, 0.315)	0.096 (−0.384, 0.574)	−0.085 (−0.482, 0.309)	MISCAN	−0.264 (−0.747, 0.221)	0.048 (−0.318, 0.413)	−0.393 (−0.789, 0.003)	−0.041 (−0.527, 0.446)	0.082 (−0.396, 0.562)
MLCI	0.303 (−0.186, 0.788)	0.102 (−0.285, 0.485)	0.200 (−0.158, 0.555)	0.288 (−0.193, 0.768)	0.309 (−0.111, 0.729)	−0.577 (−0.923, −0.233)	0.225 (−0.131, 0.580)	0.359 (−0.125, 0.840)	0.178 (−0.222, 0.575)	0.264 (−0.221, 0.747)	MLCI	0.312 (−0.058, 0.677)	−0.130 (−0.528, 0.271)	0.223 (−0.268, 0.711)	0.346 (−0.138, 0.826)
MNBI	−0.009 (−0.375, 0.359)	−0.210 (−0.423, 0.004)	−0.112 (−0.270, 0.047)	−0.023 (−0.382, 0.337)	−0.002 (−0.273, 0.270)	−0.888 (−1.015, −0.760)	−0.086 (−0.241, 0.069)	0.047 (−0.314, 0.405)	−0.134 (−0.370, 0.102)	−0.048 (−0.413, 0.318)	−0.312 (−0.677, 0.058)	MNBI	−0.441 (−0.680, −0.203)	−0.089 (−0.457, 0.280)	0.034 (−0.322, 0.392)
NBI	0.432 (0.035, 0.831)	0.231 (−0.034, 0.494)	0.329 (0.108, 0.550)	0.418 (0.027, 0.809)	0.439 (0.126, 0.752)	−0.447 (−0.647, −0.246)	0.355 (0.136, 0.574)	0.489 (0.097, 0.880)	0.307 (0.024, 0.589)	0.393 (−0.003, 0.789)	0.130 (−0.271, 0.528)	0.441 (0.203, 0.680)	NBI	0.352 (−0.048, 0.752)	0.475 (0.086, 0.866)
OEME	0.080 (−0.409, 0.566)	−0.122 (−0.507, 0.263)	−0.024 (−0.381, 0.334)	0.066 (−0.416, 0.549)	0.086 (−0.333, 0.508)	−0.799 (−1.146, −0.454)	0.002 (−0.354, 0.359)	0.136 (−0.347, 0.617)	−0.046 (−0.445, 0.352)	0.041 (−0.446, 0.527)	−0.223 (−0.711, 0.268)	0.089 (−0.280, 0.457)	−0.352 (−0.752, 0.048)	OEME	0.123 (−0.357, 0.603)
TXIIEE	−0.043 (−0.5217, 0.437)	−0.244 (−0.620, 0.131)	−0.146 (−0.494, 0.201)	−0.057 (−0.530, 0.418)	−0.036 (−0.448, 0.375)	−0.922 (−1.256, −0.588)	−0.120 (−0.468, 0.225)	0.013 (−0.461, 0.487)	−0.168 (−0.558, 0.218)	−0.082 (−0.562, 0.396)	−0.346 (−0.826, 0.138)	−0.034 (−0.392, 0.322)	−0.475 (−0.866, −0.086)	−0.123 (−0.603, 0.357)	TXIIEE

#### 2.11.4 Negative predictive value

Network Meta-analysis results demonstrated differences in negative predictive value compared to the gold standard for various endoscopic techniques: AI-BLI [MD = 0.963, 95%CI: (0.777, 1.151)], AI-LCI [MD = 0.916, 95%CI: (0.822, 1.010)], AI-WLE [MD = 0.862, 95% CI: (0.809, 0.915)], BLI [MD = 0.694, 95% CI: (0.509, 0.879)], CLE [MD = 0.913, 95% CI: (0.779, 1.046)], LCI [MD = 0.831, 95% CI: (0.780, 0.880)], M-BLI [MD = 0.980, 95% CI: (0.797, 1.163)], ME [MD = 0.910, 95% CI: (0.802, 1.017)], M-I-SCAN [MD = 0.983, 95% CI: (0.801, 1.166)], M-LCI [MD = 0.924, 95% CI: (0.736, 1.111)], M-NBI [MD = 0.914, 95% CI: (0.843, 0.985)], NBI [MD = 0.942, 95% CI: (0.835, 1.049)], OE-ME [MD = 0.906, 95% CI: (0.713, 1.099)], TXI-IEE [MD = 0.824, 95% CI: (0.637, 1.010)] (Table 6). Convergence analysis confirmed the stability of the results, as depicted in Figure 6B. The bar chart indicated the top five negative predictive values in descending order: M-BLI(0.232), TXI-IEE(0.206), AI-BLI(0.144), BLI(0.122), and M-I-SCAN(0.099; Figure 6C). Table 6 presents a comparison between these two distinct measures of detection.

**Table 6:** League table on NPV.

	AIBLI	AILCI	AIWLE	BLI	CLE	Golden standard	LCI	MBLI	ME	MISCAN	MLCI	MNBI	NBI	OEME	TXIIEE
AIBLI	AIBLI	−0.047 (−0.257, 0.163)	−0.101 (−0.296, 0.092)	−0.269 (−0.532, −0.006)	−0.051 (−0.282, 0.179)	−0.963 (−1.151, −0.777)	−0.133 (−0.328, 0.059)	0.017 (−0.244, 0.276)	−0.053 (−0.270, 0.161)	0.020 (−0.242, 0.281)	−0.039 (−0.305, 0.225)	−0.049 (−0.250, 0.150)	−0.0214 (−0.238, 0.193)	−0.058 (−0.327, 0.212)	−0.140 (−0.405, 0.123)
AILCI	0.047 (−0.163, 0.257)	AILCI	−0.054 (−0.162, 0.054)	−0.222 (−0.430, −0.014)	−0.003 (−0.168, 0.160)	−0.916 (−1.010, −0.822)	−0.085 (−0.193, 0.020)	0.064 (−0.142, 0.269)	−0.006 (−0.150, 0.137)	0.067 (−0.139, 0.272)	0.008 (−0.202, 0.217)	−0.002 (−0.120, 0.116)	0.026 (−0.116, 0.168)	−0.010 (−0.225, 0.204)	−0.092 (−0.301, 0.116)
AIWLE	0.101 (−0.092, 0.296)	0.054 (−0.054, 0.162)	AIWLE	−0.168 (−0.361, 0.026)	0.051 (−0.093, 0.195)	−0.862 (−0.915, −0.809)	−0.031 (−0.105, 0.040)	0.118 (−0.072, 0.308)	0.047 (−0.073, 0.168)	0.121 (−0.068, 0.311)	0.062 (−0.133, 0.257)	0.052 (−0.036, 0.141)	0.079 (−0.039, 0.199)	0.044 (−0.156, 0.243)	−0.039 (−0.232, 0.155)
BLI	0.269 (0.006, 0.532)	0.222 (0.014, 0.430)	0.168 (−0.026, 0.361)	BLI	0.219 (−0.011, 0.446)	−0.694 (−0.879, −0.509)	0.136 (−0.056, 0.328)	0.285 (0.026, 0.546)	0.215 (0.000, 0.430)	0.289 (0.028, 0.550)	0.229 (−0.033, 0.493)	0.220 (0.021, 0.419)	0.247 (0.033, 0.462)	0.211 (−0.057, 0.479)	0.129 (−0.134, 0.393)
CLE	0.051 (−0.179, 0.282)	0.003 (−0.160, 0.168)	−0.051 (−0.195, 0.093)	−0.219 (−0.446, 0.011)	CLE	−0.913 (−1.046, −0.779)	−0.082 (−0.225, 0.060)	0.067 (−0.159, 0.294)	−0.003 (−0.175, 0.169)	0.070 (−0.156, 0.298)	0.011 (−0.219, 0.240)	0.001 (−0.150, 0.153)	0.029 (−0.142, 0.201)	−0.007 (−0.241, 0.227)	−0.090 (−0.319, 0.141)
Golden standard	0.963 (0.777, 1.151)	0.916 (0.822, 1.010)	0.862 (0.809, 0.915)	0.694 (0.509, 0.879)	0.913 (0.779, 1.046)	GoldenStandard	0.831 (0.780, 0.880)	0.980 (0.797, 1.163)	0.910 (0.802, 1.017)	0.983 (0.801, 1.166)	0.924 (0.736, 1.111)	0.914 (0.843, 0.985)	0.942 (0.835, 1.049)	0.906 (0.713, 1.099)	0.824 (0.637, 1.010)
LCI	0.133 (−0.059, 0.328)	0.085 (−0.020, 0.193)	0.031 (−0.040, 0.105)	−0.136 (−0.328, 0.056)	0.082 (−0.060, 0.225)	−0.831 (−0.880, −0.780)	LCI	0.149 (−0.039, 0.339)	0.079 (−0.039, 0.198)	0.152 (−0.035, 0.342)	0.093 (−0.100, 0.287)	0.083 (−0.003, 0.171)	0.111 (−0.006, 0.230)	0.075 (−0.123, 0.275)	−0.007 (−0.199, 0.186)
MBLI	−0.017 (−0.276, 0.244)	−0.064 (−0.269, 0.142)	−0.118 (−0.308, 0.072)	−0.285 (−0.546, −0.026)	−0.067 (−0.294, 0.159)	−0.980 (−1.163, −0.797)	−0.149 (−0.339, 0.039)	MBLI	−0.070 (−0.283, 0.142)	0.004 (−0.255, 0.262)	−0.056 (−0.318, 0.206)	−0.066 (−0.262, 0.130)	−0.038 (−0.249, 0.173)	−0.074 (−0.339, 0.191)	−0.156 (−0.417, 0.104)
ME	0.053 (−0.161, 0.270)	0.006 (−0.137, 0.150)	−0.047 (−0.168, 0.073)	−0.215 (−0.430, −0.000)	0.003 (−0.169, 0.175)	−0.910 (−1.017, −0.802)	−0.079 (−0.198, 0.039)	0.070 (−0.142, 0.283)	ME	0.074 (−0.138, 0.286)	0.014 (−0.201, 0.230)	0.004 (−0.125, 0.133)	0.032 (−0.120, 0.184)	−0.004 (−0.225 , 0.217)	−0.086 (−0.301, 0.128)
MISCAN	−0.020 (−0.281, 0.242)	−0.067 (−0.272, 0.139)	−0.121 (−0.311, 0.068)	−0.289 (−0.550, −0.028)	−0.070 (−0.298, 0.156)	−0.983 (−1.166, −0.801)	−0.152 (−0.342, 0.035)	−0.004 (−0.262, 0.255)	−0.074 (−0.286, 0.138)	MISCAN	−0.059 (−0.320, 0.203)	−0.069 (−0.265, 0.126)	−0.042 (−0.253, 0.170)	−0.077 (−0.342, 0.188)	−0.160 (−0.421, 0.100)
MLCI	0.039 (−0.225, 0.305)	−0.008 (−0.217, 0.202)	−0.062 (−0.257, 0.133)	−0.229 (−0.493, 0.033)	−0.011 (−0.240, 0.219)	−0.924 (−1.111, −0.736)	−0.093 (−0.287, 0.100)	0.056 (−0.206, 0.318)	−0.014 (−0.230, 0.201)	0.059 (−0.203, 0.320)	MLCI	−0.010 (−0.209, 0.190)	0.018 (−0.197, 0.234)	−0.018 (−0.287, 0.249)	−0.100 (−0.365, 0.163)
MNBI	0.049 (−0.150, 0.250)	0.002 (−0.116, 0.120)	−0.052 (−0.141, 0.036)	−0.220 (−0.419, −0.021)	−0.001 (−0.153, 0.150)	−0.914 (−0.985, −0.843)	−0.083 (−0.171, 0.003)	0.066 (−0.130, 0.262)	−0.004 (−0.133, 0.125)	0.069 (−0.126, 0.265)	0.010(−0.190, 0.209)	MNBI	0.028 (−0.100, 0.156)	−0.008 (−0.213, 0.197)	−0.091 (−0.290, 0.108)
NBI	0.021 (−0.193, 0.238)	−0.026 (−0.168, 0.116)	−0.079 (−0.199, 0.039)	−0.247 (−0.462, −0.033)	−0.029 (−0.201, 0.142)	−0.942 (−1.049, −0.835)	−0.111 (−0.230, 0.006)	0.038 (−0.173, 0.249)	−0.032 (−0.184, 0.120)	0.042 (−0.170, 0.253)	−0.018 (−0.234, 0.197)	−0.028 (−0.156, 0.100)	NBI	−0.036 (−0.256, 0.184)	−0.118 (−0.332, 0.095)
OEME	0.058 (−0.212, 0.327)	0.010 (−0.204, 0.225)	−0.044 (−0.243, 0.156)	−0.211 (−0.479, 0.057)	0.007 (−0.227, 0.241)	−0.906 (−1.099, −0.713)	−0.075 (−0.275, 0.123)	0.074 (−0.191, 0.339)	0.004 (−0.217, 0.225)	0.077 (−0.188, 0.342)	0.018 (−0.250, 0.287)	0.008 (−0.197, 0.213)	0.036 (−0.184, 0.256)	OEME	−0.082 (−0.350, 0.186)
TXIIEE	0.140 (−0.123, 0.405)	0.092 (−0.116, 0.301)	0.039 (−0.155, 0.232)	−0.129 (−0.393, 0.134)	0.090 (−0.141, 0.319)	−0.824 (−1.010, −0.637)	0.007 (−0.186, 0.199)	0.156 (−0.104, 0.417)	0.086 (−0.128, 0.301)	0.160 (−0.100, 0.421)	0.100 (−0.163, 0.365)	0.091 (−0.108, 0.290)	0.118 (−0.095, 0.332)	0.082 (−0.186, 0.350)	TXIIEE

### 2.12 Regression analysis

To examine the effect of age as well as the classification of the gold standard on the results, we performed a meta-regression analysis using StataMP 18.

#### 2.12.1 Regression analysis of age

The results of regression analysis showed that the mean age of the study population was not a statistically significant moderator of SE (*p* > 0.3370), PPV (*p* > 0.1370), and NPV (*p* > 0.8860; Table 7). In addition, there is no sufficient reason to deny that the mean age of the population is not a moderator of SP (*p* > 0.0030). Table 7 provides details on the age regression analysis of the results.

**Table 7:** The age regression analysis of the results.

	Coefficient	Std. err.	*t*	*p* > |t|	95% conf. interval
SE	−0.0023	0.0023	−0.9700	0.3370	(−0.0069, 0.0024)
SP	0.0038	0.0012	3.0800	0.0030	(0.0013, 0.0063)
PPV	0.0043	0.0029	1.5100	0.1370	(−0.0014, 0.0101)
NPV	−0.0002	0.0016	−0.1400	0.8860	(−0.0035, 0.0030)

#### 2.12.2 Regression analysis of gold standard classification

Regression analysis showed that the classification of the gold standard was not a statistically significant moderator of SE (*p* > 0.4280), PPV (*p* > 0.4280) and NPV (*p* > 0.0790; Table 8). In addition, there was still no sufficient reason to reject that the classification of the gold standard was not a moderator of SP (*p* > 0.0330). Table 8 details the results of the gold standard classification regression analysis.

**Table 8:** The regression analysis for gold standard classification of results.

	Coefficient	Std. err.	*t*	*p* > |t|	95% conf. interval
SE	0.0174	0.0218	0.8000	0.4280	(−0.0264, 0.0612)
SP	0.0268	0.0123	2.1900	0.0330	(0.0022, 0.0515)
PPV	0.0219	0.0274	0.8000	0.4280	(−0.0330, 0.0768)
NPV	0.0259	0.0144	1.7900	0.0790	(−0.0031, 0.0548)

## 3 Discussion

Digestive endoscopy is a relatively invasive examination, which is the basis of all invasive examination methods of the upper digestive tract. Compared with non-invasive examination, digestive endoscopy may cause certain throat discomfort, nausea or transient digestive discomfort (Liang et al., 2022), but it can more accurately determine the scope of Hp infection and the degree of damage to the gastric mucosa, which is an advantage that other methods do not have.

This study aimed to assess the diagnostic efficacy of various novel endoscopic techniques in screening for gastric *H. pylori* infection. It encompassed 36 articles with 54 studies, incorporating 14 distinct endoscopic techniques and gold-standard detection methods. The quantitative analysis included a substantial sample size of 7,230 patients. Our findings indicate that M-BLI, M-I-SCAN, AI-BLI, and TXI-IEE exhibit higher diagnostic efficacy than the gold standard. Our study represents the first comprehensive network meta-analysis of diagnostic tests for these novel endoscopic techniques in diagnosing gastric *H. pylori* infection.

The network meta-analysis results showed that in the ranking chart of new endoscopic techniques, M-BLI ranked first in sensitivity and positive predictive value, second in negative predictive value, and fourth in specificity.

M-BLI is an innovative image enhancement technique integrating ME and BLI. ME, a magnifying endoscopy, enhances resolution by incorporating a zoom lens to aid endoscopists in better observing gastric mucosa details, including pits, collecting venules, and capillary shapes (Bessède et al., 2017). Observing gastric mucosa with *H. pylori* infection often reveals enlarged pits, irregular or vanished capillary networks, and irregular or absent collecting veins. Conversely, a honeycomb-like capillary network, gastric body RAC, and regular round pits frequently indicate the absence of *H. pylori* infection in the gastric mucosa (Qi et al., 2016). BLI (Blue Laser Imaging Endoscopy) is an advanced contrast imaging technology developed by Fujifilm Corporation, Japan. A 450 nm laser irradiates phosphor to produce illumination light similar to a xenon lamp. BLI combines an intense 410 nm laser, a weak 450 nm laser, and a fluorescent lamp for narrow-band light observation. In a study by Nishikawa et al. involving 441 patients with atrophic gastritis, BLI categorized gastric mucosa into spot, crack, and patch types. BLI demonstrated a specificity and positive predictive value of 95.3 and 86.5%, respectively, for *H. pylori* infection. Spot type may indicate *H. pylori* infection, while crack type may suggest inflammatory changes after *H. pylori* eradication (Nishikawa et al., 2018). BLI, combined with magnifying ME, can observe high-contrast images of superficial mucosal vessels with a close magnifying field of view and maintain vessel contrast by adjusting laser intensity. It facilitates high brightness and long-distance observation, enhancing the detection of delicate structures and overcoming deficiencies in certain endoscopes like NBI with a dark field of view (Kaneko et al., 2014; Osawa and Yamamoto, 2014; Yoshida et al., 2014a,b; Miyaki et al., 2015; Dohi et al., 2017). Tahara T et al. conducted a randomized controlled trial to explore the diagnostic power of M-BLI endoscopy for gastric *H. pylori* positivity in cancer-free patients, comparing the data with the diagnostic power of M-NBI endoscopy. The study included 113 patients in the M-BLI group and 112 in the M-NBI group.

The large curvature of the mid-upper body of the stomach was meticulously assessed using M-BLI or M-NBI. Small round pits with a regular honeycomb subepithelial capillary network (SECN) regularly scattered in the collecting venules were considered negative for *H. pylori* infection. Enlarged or extended pits, unclear SECN, or dense, OK, irregular blood vessels indicated *H. pylori* positivity. The sensitivity, specificity, PPV, and NPV of BLI were 0.98, 0.92, 0.93, and 0.98, respectively, compared with 0.97, 0.81, 0.87, and 0.95 in the NBI group. No significant differences were found between M-BLI and M-NBI groups (all *p* > 0.2; Tahara et al., 2017). However, with the inclusion of more recent literature in the mesh meta-analysis, our study shows that M-BLI significantly outperforms M-NBI in SE, SP, PPV, and NPV. The reason may be that M-NBI is not enough to reveal the changes in hemoglobin absorption characteristics, and the contrast and resolution of some diseases with fine structures or similar colors are limited. However, M-BLI is not limited by spectrum compared with M-NBI, and can use the biofilm interference principle to provide a wider range of biomolecular level interaction information. It has higher contrast and resolution to provide sharper images in some cases, and has lower operational dependence. This study demonstrates the high diagnostic accuracy and utility of M-BLI in diagnosing gastric *H. pylori* infection. Therefore, considering the sensitivity and positive predictive value, M-BLI exhibits superior diagnostic performance and can be recommended as a promising detection tool for gastric *H. pylori* infection.

The ranking revealed that M-I-SCAN excelled in negative predictive value, ranking first; its sensitivity and specificity were third, and positive predictive value fifth. I-SCAN developed by Pentax Company in Japan, is a computer virtual staining imaging technology with three critical functions for real-time image enhancement: surface enhancement (SE), contrast enhancement (CE), and hue enhancement (TE). The first two enhance lesion identification without significantly altering the color hue and image brightness, often used in tandem. Hue enhancement makes color, hue, and structural changes more apparent after lesion identification. TE includes modes like g for the stomach, c for the intestine, e for the esophagus, b for Barret’s esophagus, p for the mucosa, and v for the small blood vessels. Besides microvascular morphology and fine structure observation, I-Scan demonstrates multi-channel and multi-color contrast capabilities, offering unique advantages for determining lesion edges and classifying glandular tube openings (Glover et al., 2020b; Tosun et al., 2022). Sharm et al. conducted a study with 146 patients. WLE’s sensitivity, specificity, positive predictive value, negative predictive value, and accuracy in diagnosing *H. pylori* infection were 59, 100, 100, 69, and 78%, respectively. I-Scan endoscopy exhibited 100, 95, 96, 100, and 97% in the same metrics. I-Scan was superior in observing the fine structure of gastric mucosa, but additional studies are required to understand the *H. pylori* infection pattern (Sharma et al., 2017). A magnifying endoscopic ME and an i-scan have been developed, providing more explicit images of mucosal and vascular patterns. Qi et al. utilized M-I-Scan and ME to observe *H. pylori* infection in the gastric mucosa of 84 patients. The accuracy of M-I-Scan in diagnosing *H. pylori* infection (94.0% vs. 84.5%, *p* < 0.05, *p* = 0.046) and specificity (93.5% vs. 80.6%, *p* = 0.032) were higher than ME (Qi et al., 2013). Therefore, combining ME with I-SCAN testing can uphold a robust negative predictive value for diagnosing *H. pylori* infection and potentially reduce medical costs.

The ranking chart indicates that TXI-IEE secured the top position in specificity, claimed the second spot in positive predictive value, ranked ninth in sensitivity, and held the tenth position in negative predictive value. TXI-IEE, developed by Olympus Medical Systems (Tokyo, Japan) in 2020, is an innovative image enhancement endoscopy technique known as Texture and Color Enhanced Imaging. Compared to conventional white light endoscopy (WLE) images, TXI-IEE exhibits improved texture, heightened brightness, and a broader color spectrum. Consequently, TXI-IEE facilitates the detection of subtle tissue variations and color alterations in the gastric mucosa. Developed by Olympus Medical Systems (Tokyo, Japan) in 2020, TXI-IEE is a novel image-enhanced endoscopy technique specializing in texture and color-enhanced imaging. Given these enhancements over WLE, TXI-IEE may contribute to better visibility of endoscopic findings related to gastric *H. pylori* infection during routine endoscopy (Ishikawa et al., 2021; Sato, 2021). Kitagawa Y et al. retrospectively curated a set of 22 endoscopic images obtained from 60 consecutive patients using WLI and TXI-IEE, respectively. Five independent endoscopists reviewed randomly displayed image sets and evaluated endoscopic gastric *H. pylori* infection status based on the Kyoto classification of gastritis. The study also examined the association of endoscopic features with three categories of gastric *H. pylori* infection status (currently infected, previously infected, and noninfected). Results indicated that TXI-IEE exhibited significantly higher diagnostic accuracy for active gastritis than WLI (85.3% vs. 78.7%; *p* = 0.034). Odds ratios (ORs) for all endoscopy-specific features related to gastric *H. pylori* infection status were higher in the TXI-IEE group than in the WLI group. Notably, diffuse redness was the sole observation for current infection (OR, 22.0 and 56.1, respectively). Geographic redness was considered indicative of previous infection (OR 6.3 and 11.0, respectively), while regular alignment of collecting venules (RAC) was associated with an uninfected status (OR 25.2 and 42.3, respectively). All specific endoscopic features linked to gastric *H. pylori* infection status demonstrated higher ORs in the TXI-IEE group than in the WLI group. TXI-IEE enhanced the visibility of diffuse redness, geographic redness, and RAC by creating more excellent contrast (Kitagawa et al., 2023). Therefore, TXI-IEE, with its highest specificity, can potentially reduce the need for unnecessary gastric biopsy. However, it has limitations due to its low sensitivity and negative predictive value.

The ranking chart indicates that AI-BLI ranks second in sensitivity, third in positive and negative predictive values, and sixth in specificity. Artificial intelligence (AI) is the fastest-growing field in endoscopic research, which is increasingly applied in clinical practice, particularly for image recognition and classification (Cho and Bang, 2020). In contrast to optical endoscopy, AI-assisted endoscopy exhibits operator-independent characteristics, ensuring a completely objective diagnostic process. In clinical practice, AI-assisted endoscopy proves valuable for offering second opinions and reducing operator dependence in diagnostic endoscopy (Hoogenboom et al., 2020). Wu et al. demonstrated that AI, coupled with BLI, enhances innovation by identifying the typical structure of the digestive tract. This capability alerts endoscopists to missed sites, significantly reducing the blind spot rate in digestive endoscopy (Wu et al., 2019). Nakashima H et al. developed an artificial intelligence system to predict gastric *H. pylori* infection status using blue laser imaging (BLI)-bright and linked color imaging (LCI) endoscopic images. Two hundred twenty-two patients underwent WL, BLI-bright, and LCI to capture three still images of the gastric lesser curvature. Among them, 162 patients constituted the training set, while the remaining 60 patients served as the test set for verification. Results revealed that the area under the curve (AUC) of the receiver operating characteristic analysis for WLI was 0.66.

The AUC of BLI-bright and LCI were 0.96 and 0.95, respectively. The AUC of the BLI-bright and LCI groups significantly exceeded that of the WLI group (*p* < 0.01; Nakashima et al., 2018). A systematic review and network meta-analysis conducted by Bang CS et al. further demonstrated the clinical utility of AI algorithms as an additional tool for predicting gastric *H. pylori* infection during endoscopic surgery (Bang et al., 2020). The diagnostic indexes of AI-BLI are promising for use as a detection tool. Still, its diagnosis is susceptible to the endoscopic images included in the study, introducing selection bias and, therefore, certain limitations.

## 4 Advantages and limitations

Firstly, our study encompassed 36 articles, comprising 54 observational studies, exploring 14 new endoscopic techniques, and involving 7,230 patients undergoing these novel techniques for diagnosing gastric *H. pylori* infection. The study stands out for its extensive literature coverage, substantial sample size, minimal heterogeneity in results, and rigorous methodology. Secondly, our research and its foundational studies confront certain limitations. Some newly developed endoscopic diagnostic techniques received limited coverage in the literature. The endoscopic operator’s experience might influence the efficacy of specific diagnostic methods. The inclusion of patients may have been impacted by factors such as age or medications taken, contributing to diagnostic variations among different gold standards. Readers should exercise caution in interpreting our study’s results. For instance, only one report exists on M-BLI, M-I-SCAN, TXI-IEE, and AI-BLI for diagnosing gastric *H. pylori* infection, indicating a need for further expansion and exploration.

## 5 Conclusion

In this study, we comprehensively compared 14 novel endoscopic techniques with the gold standard for diagnosing gastric *H. pylori* infection, utilizing Bayesian network meta-analysis. The findings indicate that M-BLI and M-I-SCAN exhibit robust diagnostic performance, emerging as particularly suitable endoscopic techniques for diagnosing gastric *H. pylori*. Despite some limitations, TXI-IEE and AI-BLI serve as valuable tools for early detection and diagnosis of gastric *H. pylori* infection, holding clinical significance in minimizing unnecessary biopsies and optimizing medical resource utilization. Nevertheless, this conclusion warrants validation through additional literature, and future research demands more meticulously designed, large-scale, and multicenter studies to further elucidate the application value of various new endoscopic techniques in diagnosing patients with gastric *H. pylori* infection.

## Data Availability

The original contributions presented in the study are included in the article/Supplementary material, further inquiries can be directed to the corresponding author.
