# Supplementary figures and images for: Novel endoscopic techniques for the diagnosis of gastric Helicobacter pylori infection: a systematic review and network meta-analysis
Source: Front Microbiol. 2024 Aug 26;15:1377541. doi: 10.3389/fmicb.2024.1377541 (PMC11404567; doi:10.3389/fmicb.2024.1377541)

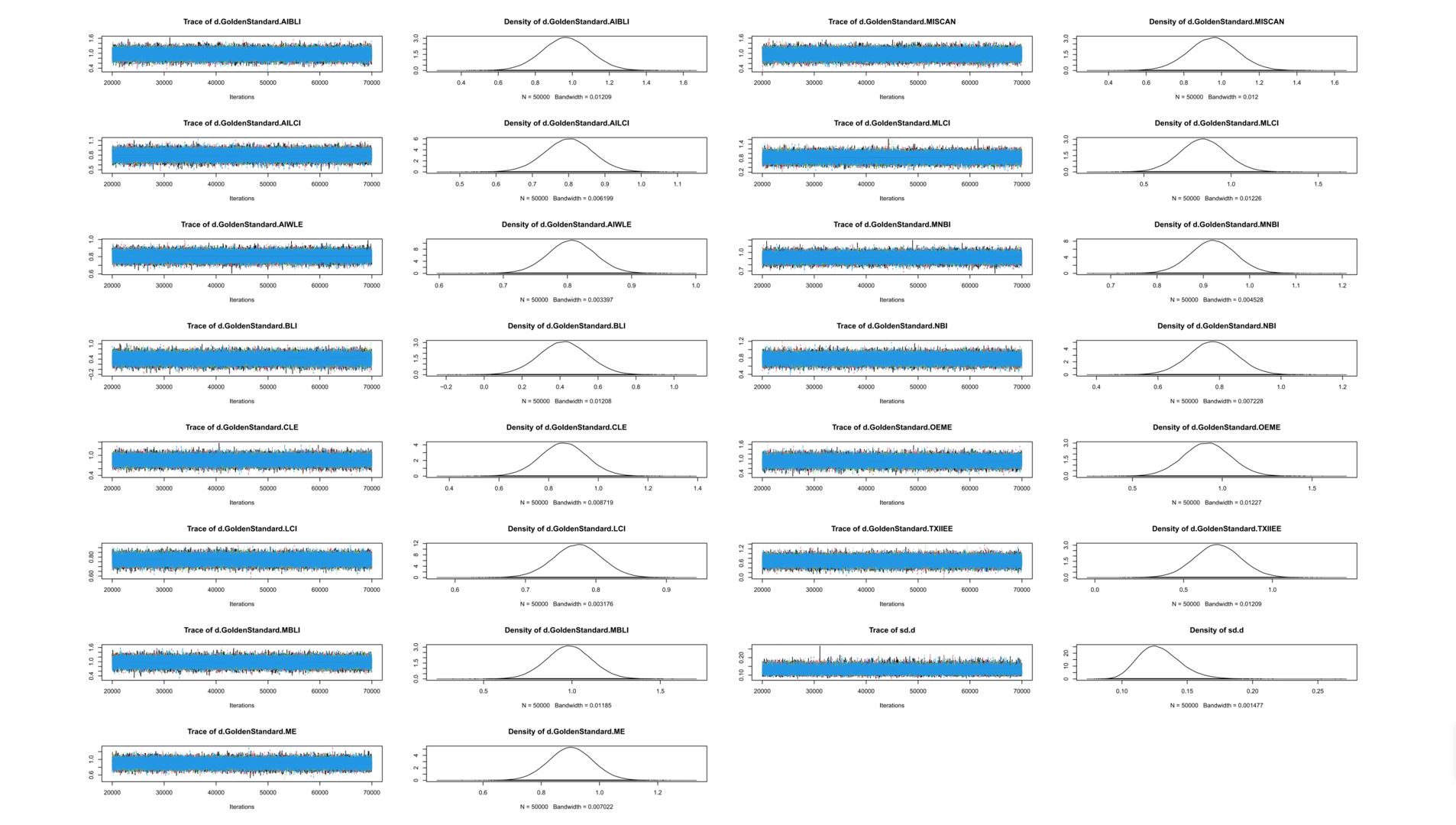

Supplement: Supplementary file 1 [file Image_1.JPEG]

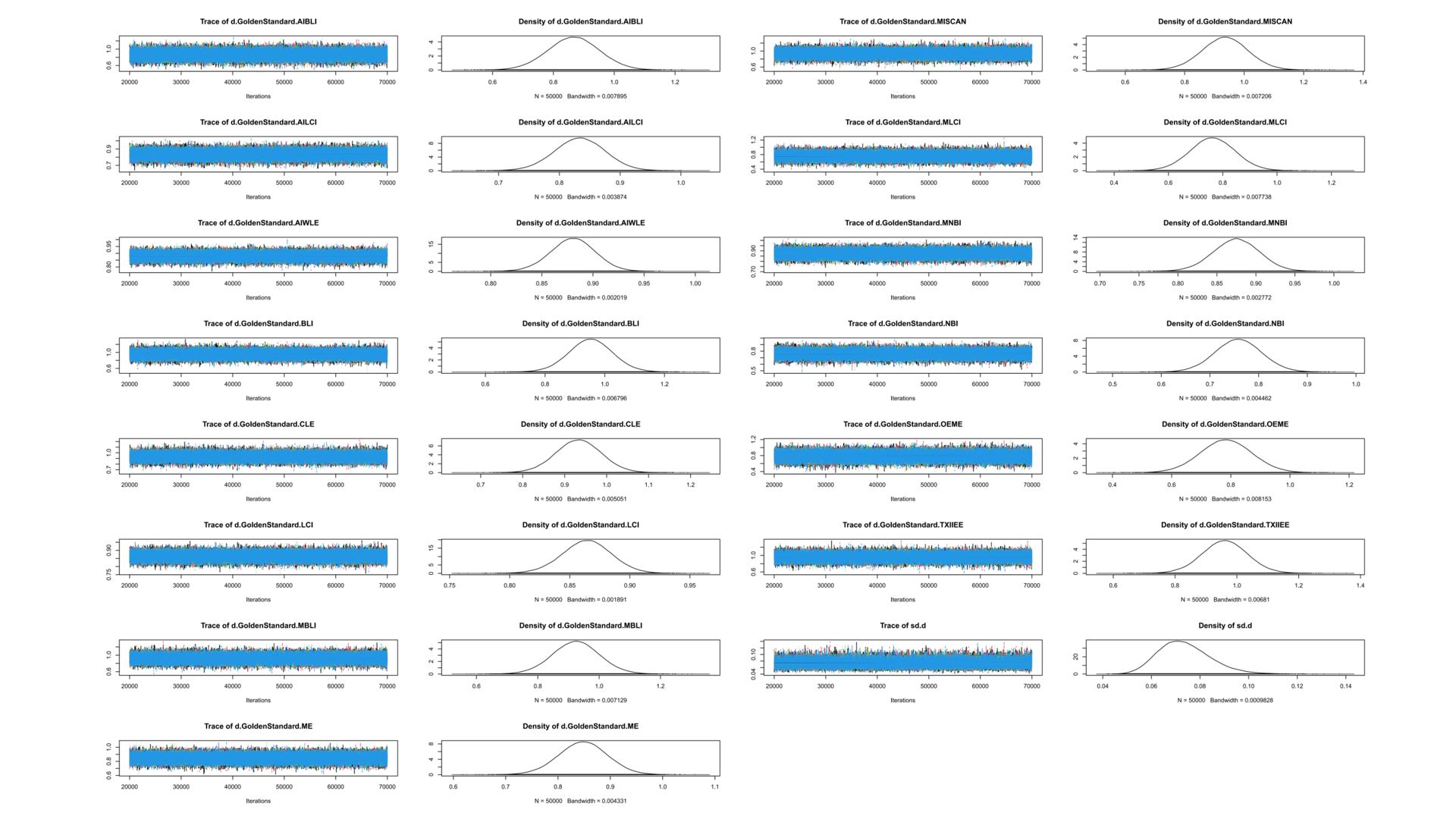

Supplement: Supplementary file 2 [file Image_2.JPEG]

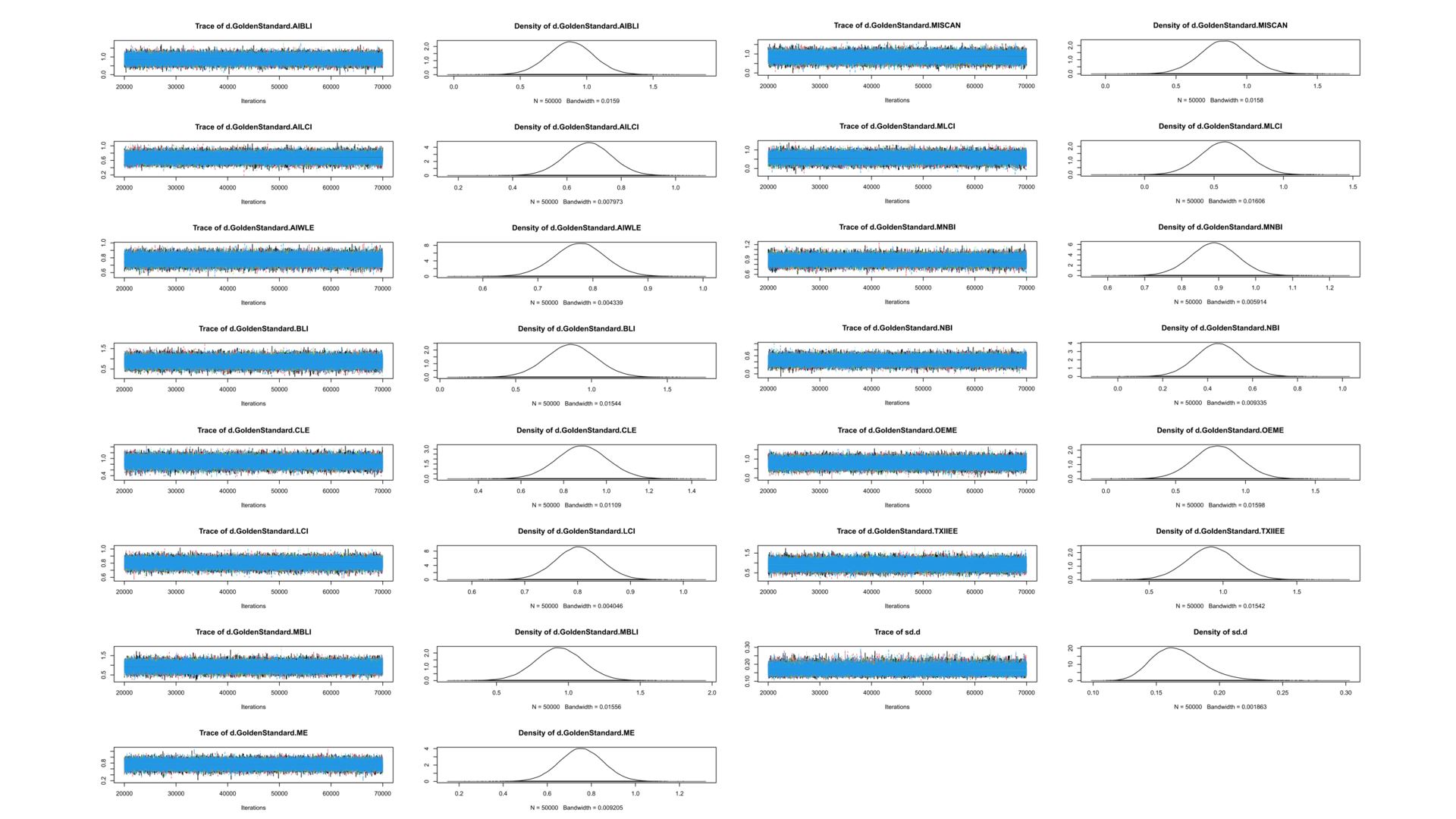

Supplement: Supplementary file 3 [file Image_3.JPEG]

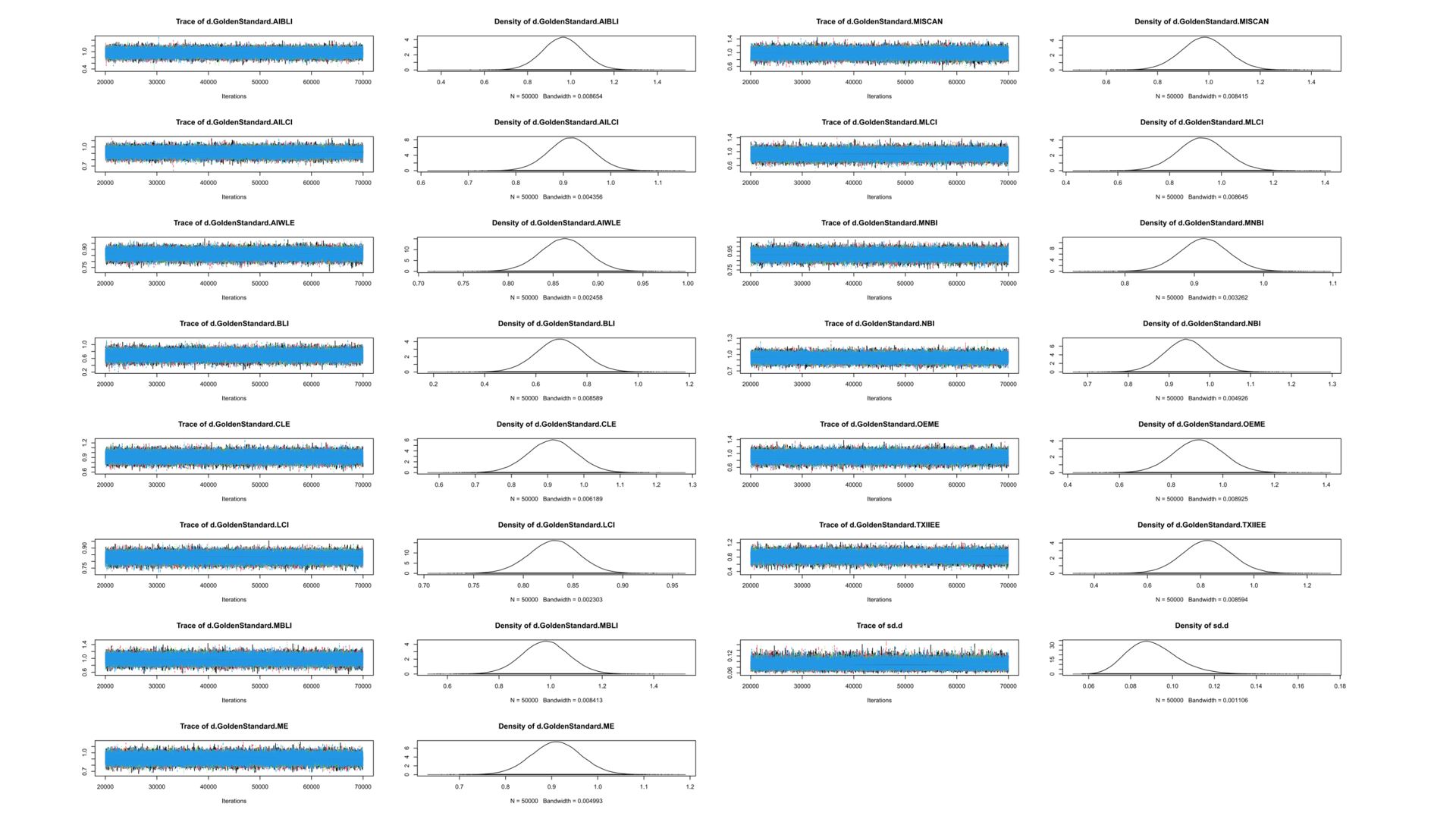

Supplement: Supplementary file 4 [file Image_4.JPEG]
