# Supplementary material for: Novel endoscopic techniques for the diagnosis of gastric Helicobacter pylori infection: a systematic review and network meta-analysis
Source: Front Microbiol. 2024 Aug 26;15:1377541. doi: 10.3389/fmicb.2024.1377541 (PMC11404567; doi:10.3389/fmicb.2024.1377541)
Supplement: Supplementary file 5 [file Table_1.DOCX]

Supplementary Material

# Supplementary Tables

For more information on Supplementary Material and for details on the different file types accepted, please see [here](https://www.frontiersin.org/guidelines/author-guidelines#supplementary-material).

**
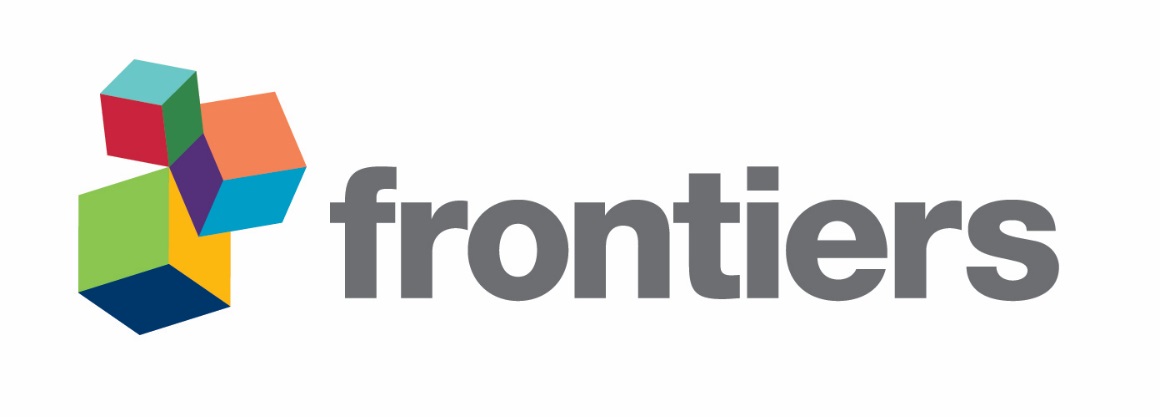
**

**Supplementary Table 1.**

**Supplementary Table 1**Search strategy on Web Of Science.

| #1 | (((((ALL=(Helicobacter pylori)) OR ALL=(Helicobacter nemestrinae)) OR ALL=(Campylobacter pylori)) OR ALL=(Campylobacter pylori subsp. pylori)) OR ALL=(Campylobacter pyloridis)) OR ALL=(H.pylori) |
| --- | --- |
| #2 | ALL=(Magnifying endoscope) |
| #3 | #1AND #2 |
| #4 | (((((((((ALL=(Narrow Band Imaging)) OR ALL=(Band Imaging, Narrow)) OR ALL=(Band Imagings, Narrow)) OR ALL=(Imaging, Narrow Band)) OR ALL=(Imagings, Narrow Band)) OR ALL=(Narrow Band Imagings)) OR ALL=(Narrowband Imaging)) OR ALL=(Imaging, Narrowband)) OR ALL=(Imagings, Narrowband)) OR ALL=(Narrowband Imagings) |
| #5 | #1 AND #4 |
| #6 | ALL=(Blue laser imaging) |
| #7 | #1AND #6 |
| #8 | ALL=(Linked Color Imaging) |
| #9 | #1AND #8 |
| #10 | ALL=(Fuji Intelligent Chromo) |
| #11 | #1 AND #10 |
| #12 | (ALL=(Iscan)) OR ALL=(I-Scan) |
| #13 | #1 AND #12 |
| #14 | (((((((((((((((((ALL=(Microscopy, Confocal)) OR ALL=(Confocal Microscopy)) OR ALL=(Confocal Microscopies)) OR ALL=(Microscopies, Confocal)) OR ALL=(Laser Scanning Microscopy)) OR ALL=(Laser Scanning Microscopies)) OR ALL=(Microscopies, Laser Scanning)) OR ALL=(Microscopy, Laser Scanning)) OR ALL=(Scanning Microscopies, Laser)) OR ALL=(Scanning Microscopy, Laser)) OR ALL=(Microscopy, Confocal, Laser Scanning)) OR ALL=(Laser Scanning Confocal Microscopy)) OR ALL=(Confocal Laser Scanning Microscopy)) OR ALL=(Confocal Microscopy, Scanning Laser)) OR ALL=(Laser Microscopy)) OR ALL=(Laser Microscopies)) OR ALL=(Microscopies, Laser)) OR ALL=(Microscopy, Laser) |
| #15 | #1 AND #14 |
| #16 | (((((((ALL=(Spectrum Analysis, Raman)) OR ALL=(Raman Spectrum Analysis)) OR ALL=(Raman Spectroscopy)) OR ALL=(Spectroscopy, Raman)) OR ALL=(Analysis, Raman Spectrum)) OR ALL=(Raman Optical Activity Spectroscopy)) OR ALL=(Raman Scattering)) OR ALL=(Scattering, Raman) |
| #17 | #1 AND #16 |
| #18 | (((((((((ALL=(Optical Imaging)) OR ALL=(Imaging, Optical)) OR ALL=(Fluorescence Imaging)) OR ALL=(Imaging, Fluorescence)) OR ALL=(Fundus Autofluorescence Imaging)) OR ALL=(Autofluorescence Imaging, Fundus)) OR ALL=(Fundus Autofluorescence Imagings)) OR ALL=(Imaging, Fundus Autofluorescence)) OR ALL=(Autofluorescence Imaging)) OR ALL=(Imaging, Autofluorescence) |
| #19 | #1 AND #18 |
| #20 | (((((((((((((((((((((((((((ALL=(Neural Networks, Computer)) OR ALL=(Computer Neural Network)) OR ALL=(Computer Neural Networks)) OR ALL=(Network, Computer Neural)) OR ALL=(Networks, Computer Neural)) OR ALL=(Neural Network, Computer)) OR ALL=(Models, Neural Network)) OR ALL=(Model, Neural Network)) OR ALL=(Network Model, Neural)) OR ALL=(Network Models, Neural)) OR ALL=(Neural Network Model)) OR ALL=(Neural Network Models)) OR ALL=(Computational Neural Networks)) OR ALL=(Computational Neural Network)) OR ALL=(Network, Computational Neural)) OR ALL=(Networks, Computational Neural)) OR ALL=(Neural Network, Computational)) OR ALL=(Neural Networks, Computational)) OR ALL=(Perceptrons)) OR ALL=(Perceptron)) OR ALL=(Connectionist Models)) OR ALL=(Connectionist Model)) OR ALL=(Model, Connectionist)) OR ALL=(Models, Connectionist)) OR ALL=(Neural Networks (Computer))) OR ALL=(Network, Neural (Computer))) OR ALL=(Networks, Neural (Computer))) OR ALL=(Neural Network (Computer)) |
| #21 | #1 AND #20 |
| #22 | (((((ALL=(Diagnosis, Computer-Assisted)) OR ALL=(Diagnosis, Computer Assisted)) OR ALL=(Computer-Assisted Diagnosis)) OR ALL=(Computer Assisted Diagnosis)) OR ALL=(Computer-Assisted Diagnoses)) OR ALL=(Diagnoses, Computer-Assisted) |
| #23 | #1 AND #22 |
| #24 | (((((((((((((((((((ALL=(Artificial Intelligence)) OR ALL=(Intelligence, Artificial)) OR ALL=(Computational Intelligence)) OR ALL=(Intelligence, Computational)) OR ALL=(Machine Intelligence)) OR ALL=(Intelligence, Machine)) OR ALL=(Computer Reasoning)) OR ALL=(Reasoning, Computer)) OR ALL=(AI (Artificial Intelligence))) OR ALL=(Computer Vision Systems)) OR ALL=(Computer Vision System)) OR ALL=(System, Computer Vision)) OR ALL=(Systems, Computer Vision)) OR ALL=(Vision System, Computer)) OR ALL=(Vision Systems, Computer)) OR ALL=(Knowledge Acquisition (Computer))) OR ALL=(Acquisition, Knowledge (Computer))) OR ALL=(Knowledge Representation (Computer))) OR ALL=(Knowledge Representations (Computer))) OR ALL=(Representation, Knowledge (Computer)) |
| #25 | #1 AND #24 |
| #26 | #3 AND #5 AND #7 AND #9 AND #11 AND #13 AND #15 AND #17 AND #19 AND #21 AND #23 AND #25 |

**Supplementary Table 2.**

**Supplementary Table 2.** Search strategy on Embase.

| #1 | helicobacter AND pylori OR (helicobacter AND nemestrinae) OR (campylobacter AND pylori) OR (campylobacter AND subsp. AND pylori) OR (campylobacter AND pyloridis) OR h.pylori |
| --- | --- |
| #2 | magnifying AND endoscope |
| #3 | #1 AND #2 |
| #4 | narrow AND band AND imaging OR (band AND imaging, AND narrow) OR (band AND imagings, AND narrow) OR (imaging, AND narrow AND band) OR (imagings, AND narrow AND band) OR (narrow AND band AND imagings) OR (narrowband AND imaging) OR (imaging, AND narrowband) OR (imagings, AND narrowband) OR (narrowband AND imagings) |
| #5 | #1 AND #4 |
| #6 | blue AND laser AND imaging |
| #7 | #1 AND #6 |
| #8 | linked AND color AND imaging |
| #9 | #1 AND #8 |
| #10 | fuji AND intelligent AND chromo AND endoscopy |
| #11 | #1 AND #10 |
| #12 | 'i scan' OR iscan |
| #13 | #1 AND #12 |
| #14 | microscopy, AND confocal OR (confocal AND microscopy) OR (confocal AND microscopies) OR (microscopies, AND confocal) OR (laser AND scanning AND microscopy) OR (laser AND scanning AND microscopies) OR (microscopies, AND laser AND scanning) OR (microscopy, AND laser AND scanning) OR (scanning AND microscopies, AND laser) OR (scanning AND microscopy, AND laser) OR (microscopy, AND confocal, AND laser AND scanning) OR (laser AND scanning AND confocal AND microscopy) OR (confocal AND laser AND scanning AND microscopy) OR (confocal AND microscopy, AND scanning AND laser) OR (laser AND microscopy) OR (laser AND microscopies) OR (microscopies, AND laser) OR (microscopy, AND laser) |
| #15 | #1 AND #14 |
| #16 | spectrum AND analysis, AND raman OR (raman AND spectrum AND analysis) OR (raman AND spectroscopy) OR (spectroscopy, AND raman) OR (analysis, AND raman AND spectrum) OR (raman AND optical AND activity AND spectroscopy) OR (raman AND scattering) OR (scattering, AND raman) |
| #17 | #1 AND #16 |
| #18 | optical AND imaging OR (imaging, AND optical) OR (fluorescence AND imaging) OR (imaging, AND fluorescence) OR (fundus AND autofluorescence AND imaging) OR (autofluorescence AND imaging, AND fundus) OR (fundus AND autofluorescence AND imagings) OR (imaging, AND fundus AND autofluorescence) OR (autofluorescence AND imaging) OR (imaging, AND autofluorescence) |
| #19 | #1 AND #18 |
| #20 | neural AND networks, AND computer OR (computer AND neural AND network) OR (computer AND neural AND networks) OR (network, AND computer AND neural) OR (networks, AND computer AND neural) OR (neural AND network, AND computer) OR (models, AND neural AND network) OR (model, AND neural AND network) OR (network AND model, AND neural) OR (network AND models, AND neural) OR (neural AND network AND model) OR (neural AND network AND models) OR (computational AND neural AND networks) OR (computational AND neural AND network) OR (network, AND computational AND neural) OR (networks, AND computational AND neural) OR (neural AND network, AND computational) OR (neural AND networks, AND computational) OR perceptrons OR perceptron OR (connectionist AND models) OR (connectionist AND model) OR (model, AND connectionist) OR (models, AND connectionist) OR (neural AND networks AND computer) OR (network, AND neural AND computer) OR (networks, AND neural AND computer) OR (neural AND network AND computer) |
| #21 | #1 AND #20 |
| #22 | diagnosis, AND 'computer assisted' OR (diagnosis, AND computer AND assisted) OR ('computer assisted' AND diagnosis) OR (computer AND assisted AND diagnosis) OR ('computer assisted' AND diagnoses) OR (diagnoses, AND 'computer assisted') |
| #23 | #1 AND #22 |
| #24 | artificial AND intelligence OR (intelligence, AND artificial) OR (computational AND intelligence) OR (intelligence, AND computational) OR (machine AND intelligence) OR (intelligence, AND machine) OR (computer AND reasoning) OR (reasoning, AND computer) OR (ai AND artificial AND intelligence) OR (computer AND vision AND systems) OR (computer AND vision AND system) OR (system, AND computer AND vision) OR (systems, AND computer AND vision) OR (vision AND system, AND computer) OR (vision AND systems, AND computer) OR (knowledge AND acquisition AND computer) OR (acquisition, AND knowledge AND computer) OR (knowledge AND representation AND computer) OR (knowledge AND representations AND computer) OR (representation, AND knowledge AND computer) |
| #25 | #1 AND #24 |
| #26 | #3 AND #5 AND #7 AND #9 AND #11 AND #13 AND #15 AND #17 AND #19 AND #21 AND #23 AND #25 |
